# Reversible Stiffening of Biopolymeric Hybrid Networks by Dynamically Switching Cross-Links *In Situ*

**DOI:** 10.1021/acsami.5c03419

**Published:** 2025-05-13

**Authors:** Jana T. Reh, Sebastian Voigt, Leonard R. Gareis, Ufuk Gürer, Stephan A. Sieber, Berna Özkale, Oliver Lieleg

**Affiliations:** † School of Engineering and Design, Department of Materials Engineering, 9184 Technical University of Munich, Boltzmannstraße 15, Garching 85748, Germany; ‡ Center for Protein Assemblies (CPA), Technical University of Munich, Ernst-Otto-Fischer Straße 8, Garching 85748, Germany; § Munich Institute of Biomedical Engineering, Technical University of Munich, Boltzmannstraße 11, Garching 85748, Germany; ∥ TUM School of Natural Sciences, Department of Bioscience, Chair of Organic Chemistry II, Technical University of Munich (TUM), Ernst-Otto-Fischer-Str. 8, Garching 85748, Germany; ⊥ Microrobotic Bioengineering Lab (MRBL), Department of Electrical Engineering, School of Computation, Information and Technology, Technical University of Munich,Hans-Piloty-Straße 1, Garching 85748, Germany; # Munich Institute of Robotics and Machine Intelligence, Technical University of Munich, Georg-Brauchle-Ring 60, Munich 80992, Germany

**Keywords:** reversible cross-links, biopolymer, tunable stiffness, hydrogels, antibacterial

## Abstract

Achieving reversible stiffening of biopolymer networks in a controlled manner remains a challenging topic in materials science, especially when trying to assess the following changes in mechanical material properties in real time. To address these challenges, we here utilize a custom-made measurement setup that allows us to manipulate the cross-linking state of alginate-based hydrogels *in situ* while quantifying the achieved alterations in the viscoelastic response of the biopolymer networks. Interpolymer connections in the biopolymer networks are created by a combination of light-induced, covalent cross-links, ionic cross-links, and DNA-based cross-links, where the latter two can be successfully removed again by employing either chelating agents (*e*.*g*., ethylenediaminetetraacetic acid and citrate) or suitable displacement DNA strands. In part, this range of the different cross-linking options mentioned is *inter alia* made possible by incorporating the glycoprotein mucin into the alginate system, which also allows for a range of different starting (∼0.2–400 Pa), intermediate (∼25 Pa–1.6 kPa), and final stiffnesses (∼4 Pa–1.2 kPa) of the mixed hydrogel matrix. At the same time, the presence of mucins (1–4% (w/v)) in the biopolymer mixture enhances the properties of the cytocompatible hydrogel by improving its antibacterial characteristics. Such well-controllable alginate/mucin networks with dynamically switchable mechanical properties will likely find broad applications in cell cultivation studies or tissue engineering applications.

## Graphical abstract

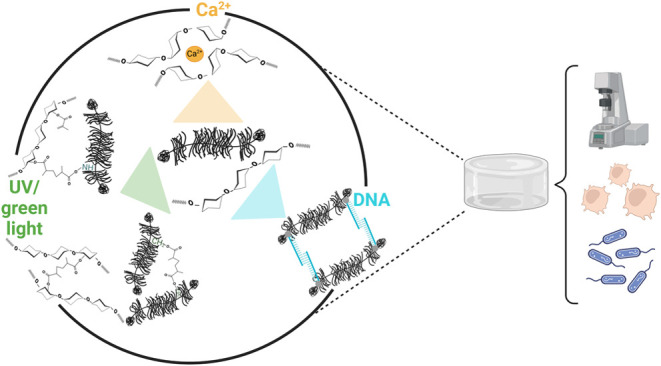

## Introduction

Even though the (bio)­polymer content in hydrogels, *i*.*e*., three-dimensional polymer networks containing large amounts of water or physiological fluid, is typically quite low (in the range of a few weight percent), they can exhibit a broad range of characteristics.^1,2^ In part, this is due to the large variety of polymers available to create hydrogel systems, and those can either be synthetic or of natural origin. Applications of such hydrogels range from drug delivery over tissue engineering to wound dressings.^3,4^ To meet the specific requirements of a biomedical application (such as biocompatibility, degradability, or dedicated mechanical properties), many different base materials and combinations created thereof have been explored.^2^ For biomedical implementations, a range of different biopolymers is available to create hydrogels (see Supporting Information, Section S1), and such biopolymers appear to be especially advantageous due to their inherently great biocompatibility, their ability to interact with other biomolecules, and their (often) high similarity to components of the extracellular matrix.^5^ However, tailoring the mechanical properties of hydrogels remains a challenge, as most biopolymer-based materials stabilized by physical entanglements alone tend to be rather soft.^6^ Accordingly, cross-linking (based on physical or chemical approaches) is typically employed to create biopolymer-based hydrogels with different stiffnesses,^7,8^ and ionic as well as covalent cross-links are the most frequently used examples.^9–11^

For certain biological applications where dynamic properties are required, however, being able to create a homogeneously cross-linked system with well-defined but permanent mechanical properties is unsatisfactory as hydrogels with switchable properties are required.^12^ In such cases, adjusting the concentration or molecular weight of the biopolymer component or the cross-linking agent prior to hydrogel preparation^13–16^ is not sufficient. To create a system with dynamically changeable mechanical properties, Rijns et al.^17^ developed a benzene-1,3,5-tricarboxamide based material into which they incorporated a poly­(*N*-isopropylacrylamide)-functionalized component to enable temperature induced changes in stiffness. Here, the system exhibits a strong increase in stiffness at around 20 °C; however, this critical temperature is not really suitable for biomedical applications, where cells are incorporated into the material, which requires the temperature to be maintained at physiological levels. Li et al.^18^ embedded magnetic microparticles into polyacrylamide hydrogels to achieve a magnetically adjustable surface stiffness that influences cellular behavior. Also, this approach is only useful for selected applications as a strong magnet needs to be brought into close contact with the material—and this is not always feasible. Other approaches make use of changes in sample pH,^19^ DNA-based cross-linking,^20,21^ or induce covalent cross-links based on photoreactions^22,23^ to trigger changes in the hydrogel stiffness.

In other approaches, several cross-linking mechanisms were combined to increase the options of how to change the hydrogel stiffness. For instance, Bian et al.^24^ changed the mechanical properties of a calmodulin-based hydrogel in two steps: first, by cross-linking with Ca^2+^ ions, which then further interacted with trifluoperazine to generate a secondary cross-linking effect. Similarly, Li et al.^25^ combined enzymatic with light-induced cross-linking strategies in gelatin hydrogels, and sequential stiffness changes in alginate hydrogels were achieved by combining ionic and covalent cross-linking strategies (the latter of which made use of illumination with green light^26^ or UV light^27^). In addition, Moheimani et al.^28^ suggested repeated autoclaving of alginate hydrogels to sequentially change the viscoelastic properties of the material. Alginate-based hydrogels provide several advantages compared to other biopolymer gels, and examples include the absence of a strong temperature dependency (which is a typical feature of, *e*.*g*., gelatin gels^29^ and can introduce undesired handling issues), good solubility in water (which is in contrast to, *e*.*g*., cellulose and its derivates^30,31^ ), and their low cost (see Supporting Information, Section S1 and Table S1).

Using pure alginate hydrogels, however, comes with some disadvantages such as problems with their long-term mechanical stability (especially when relying on ionic cross-links only^32^), insufficient controllability of degradation (when using alginate of high molecular weight^33^ and/or ionic cross-links^34^), or the lack of naturally occurring cellular binding sites.^35,36^ To mitigate these issues, incorporating another biopolymer component could be a good strategy, thus creating a composite material with a broader range of properties. For such a purpose, biopolymers like gelatin, cellulose, or fibronectin can be useful candidates. For instance, Tansik and Stowers^37^ sequentially cross-linked a composite hydrogel comprising alginate and methacrylated gelatin, and Lee et al.^38^ employed two different ions, Ca^2+^ and Fe^3+^, to create a sequential increase in stiffness in alginate/carboxymethyl cellulose beads. Moreover, Trujillo et al.^39^ created alginate/fibronectin hybrid hydrogels stabilized by a combination of ionic and covalent cross-links. There, permanent, covalent cross-links were created by illumination with UV light, whereas the ionic cross-links were dynamically switched off and on by employing a chelating agent. In detail, the cross-links were removed by washing out the ionic cross-linkers (*e*.*g*., by macroscopic immersion of the samples in phosphate buffered saline (PBS) or ethylenediaminetetraacetic acid (EDTA) solutions^39^). In a recent approach by Scott et al.,^40^ the diffusion of poly­(ethylene glycol) (PEG) into and out of an alginate-based material resulted in reversible changes in stiffness over the course of several days. By adapting the PEG concentration in the incubation medium, a dynamically switchable process was obtained. Even though switchable mechanical properties were demonstrated in these studies, they relied on very long incubation times (several hours to days) as well as end point measurements and thus could not report the kinetics of the switching processes. However, such information would be highly desirable to better understand the applicability of the different strategies in such settings, where the time window for hydrogel manipulation is limited.

Here, we address this issue by following the dynamic alteration of the viscoelastic hydrogel properties *in situ*, *i*.*e*., we follow the kinetics of processes that establish or remove cross-links in biopolymer networks. Moreover, we present a novel composite hydrogel system, in which we integrate mucin glycoproteins as a biologically active component into alginate hydrogels. With this additional biopolymer component, we leverage the intrinsic advantages of mucins, *inter alia* the presence of additional chemical moieties for attaching cross-linking motifs^41^ and its antibacterial properties.^42^ For such mucin/alginate hybrid networks, we assess the suitability of different binary combinations of covalent, ionic, and DNA-based cross-linking strategies and assess their reversibility. In addition, we investigate the biocompatibility of the components used to generate the different cross-linked systems and demonstrate the antibacterial effect brought about by the mucins toward several bacterial strains.

## Results and Discussion

We here aim at creating a mixed network of interpenetrating biopolymers (*i*.*e*., alginate and mucin) that is stabilized by a combination of different cross-links; those cross-links should be activatable or inactivatable on demand. The range of cross-links explored here is summarized in Figure 1. It comprises ionic cross-links, covalent cross-links (that can be generated between methacrylated macromolecules upon light exposure in the presence of a photoinitiator), and transient cross-links established by partially self-complementary DNA strands that are conjugated to the biopolymers. Whereas the ionic and DNA-based cross-links should be (partially) removable by adding a chelator and a displacement DNA strand, respectively, the covalent cross-links will—of course—be permanent once they have been created.

**Figure 1:**
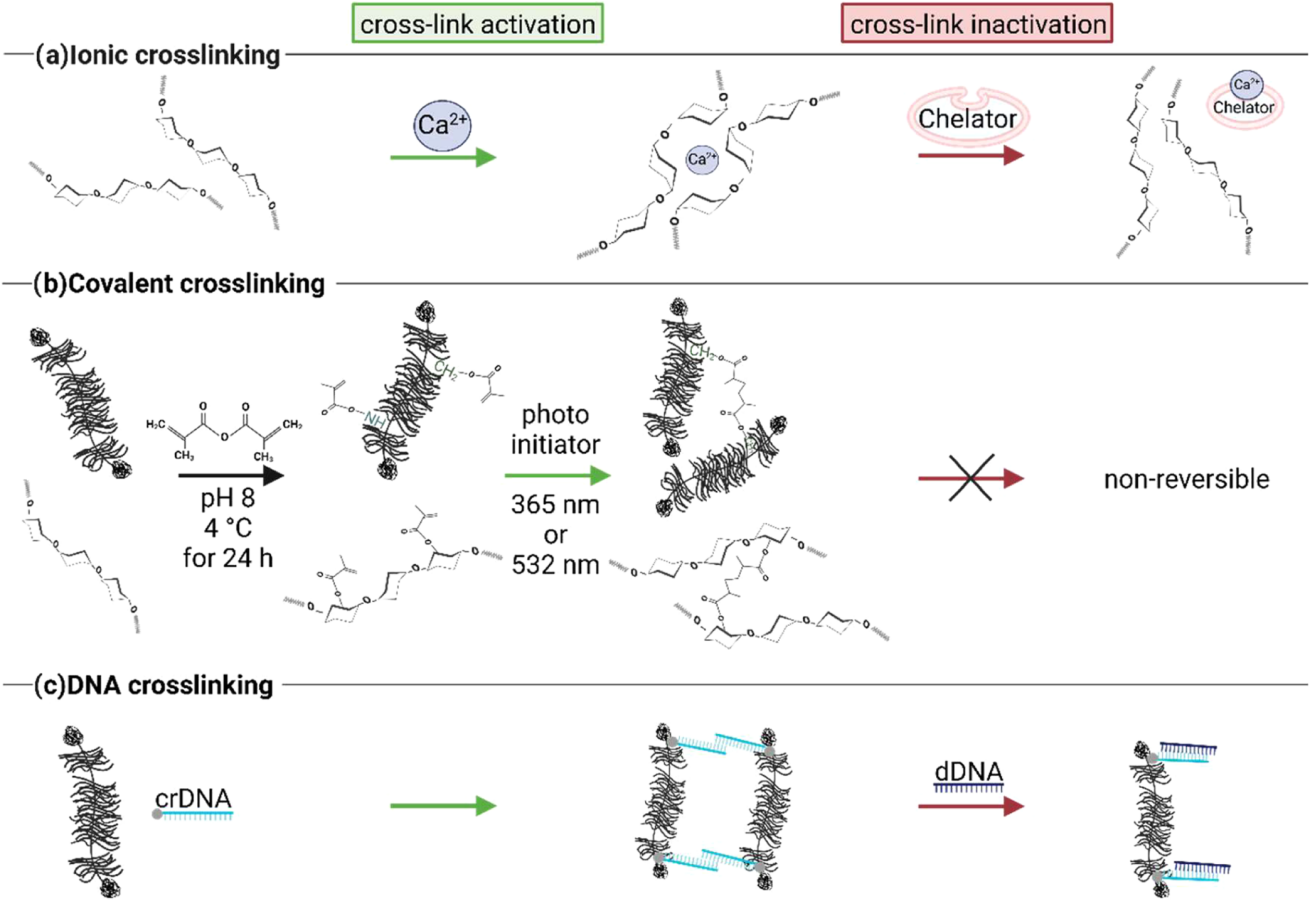
Schematic visualization of different options to create activatable/inactivatable cross-links between biopolymers. These comprise (a) ionic cross-linking, which should be reversable by adding a chelator; (b) nonreversible, covalent cross-linking of methacrylated biopolymers; (c) DNA assisted cross-linking, which should be reversable by adding a suitable displacement DNA strand. The schematic drawings were made using BioRender: https://BioRender.com/o69y852.

To be able to manipulate cross-links in a biopolymer hydrogel sample *in situ*, we make use of a dedicated measuring setup, which was previously developed in our lab.^43^ With this setup, it is possible to add reactants to a sample during the measurement by allowing for a diffusive exchange of small molecules between the sample and a reservoir located below. This enables us to follow the dynamic alteration of material properties in response to such a manipulation. The diffuse entry of reactants into the sample occurs through a macroporous bottom plate that is covered with a microporous membrane (see Materials and Methods for details). In addition, to allow for an *in situ* formation of covalent cross-links between distinct biopolymers, this setup is further modified by replacing the (nontransparent) standard measuring geometry provided by the device manufacturer with an in-house crafted version that is transparent toward UV light (see Supporting Information, Section S2). With this modification, it is possible to illuminate a sample either from above (which enables the simultaneous use of the porous bottom plate) or from below (by employing a transparent bottom plate that is commercially available from Anton Paar). And indeed, when inducing the UV-triggered cross-linking of methacrylated alginate (mAlg) or methacrylated mucin (mMUC) samples in the presence of a suitable photoinitiator, we obtain very similar gelation kinetics as well as final gel stiffnesses with either illumination method (see Figure 2a): In the case of 4% (w/v) mAlg, illumination from below results in a gel stiffness around 600 Pa, whereas illumination from above yields a gel with a stiffness of ∼300 Pa. For 2% (w/v) mMUC, positioning the UV light below the sample induces a gel stiffness of around 30 and ∼20 Pa is achieved via illumination from above.

**Figure 2:**
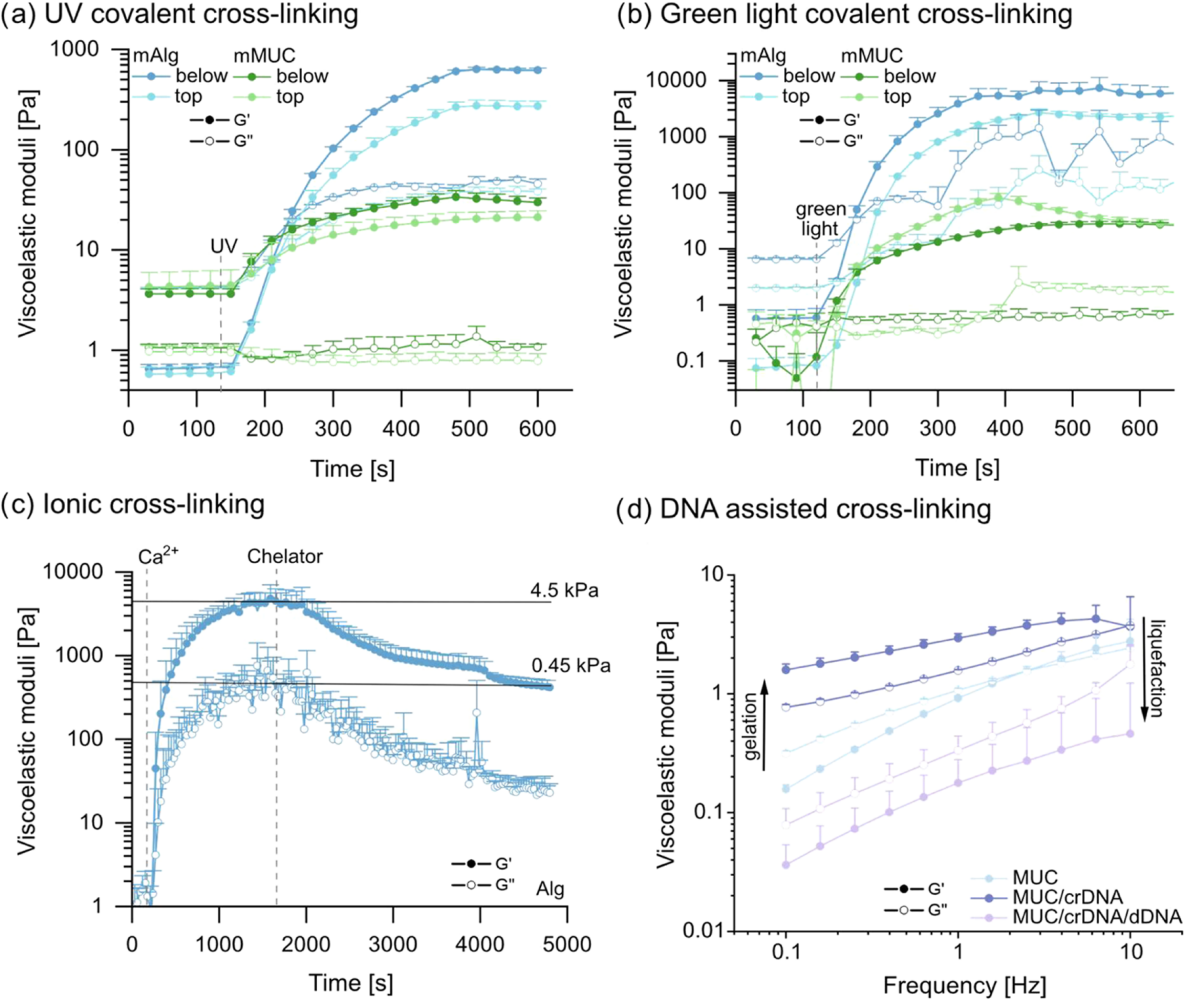
Viscoelastic properties of pure alginate or pure mucin systems upon the activation/deactivation of cross-links *in situ*. 4% (w/v) mAlg or 2% (w/v) mMUC samples were cross-linked by illumination with UV light (a) or with green light (b) from below or from above. (c) 4% (w/v) alginate (Alg) was first ionically cross-linked with Ca^2+^ ions and then exposed to a chelator mix comprising 0.5 M EDTA and 0.25 M citrate. (d) frequency sweeps obtained for a pure mucin (MUC) 2% (w/v) solution, a MUC 2% (w/v) network cross-linked with 30 μM crDNA, and a MUC 2% (w/v) sample containing both, 30 μM crDNA as well as 200 μM dDNA. Data shown represents mean values, error bars depict the standard deviation as calculated from *n* = 3 samples.

As UV light might not be suitable for every possible application of the envisioned alginate/mucin hybrid gels (*e*.*g*., for studies involving cells, which are sensitive to UV light), we also test an alternative light-induced cross-linking strategy employing a green light source (see Figure 2b).

Again, mMUC and mAlg samples were cross-linked in the presence of a suitable cross-linking agent by illumination from above or from below. Here, exposure of an mAlg solution to green light induces gelation with kinetics similar to what we obtained for UV-based cross-linking, and the generated gels have stiffness values around 6 kPa for illumination from below and ∼3.5 kPa for illumination from above. For mMUC solutions, the created gels have stiffnesses around 30 Pa for either illumination direction. In other words, also when employing green light, both strategies of exposing the material to light to induce cross-linking are similarly successful. In this case, whereas the stiffness values obtained for cross-linked mMUC gels are in a similar range for both types of light sources, cross-linking mAlg by employing green light results in higher stiffnesses compared to when UV light is used. However, when conducting this direct comparison, it is important to note that the two light sources have different intensities, and the employed cross-linking agents differ in efficiency and (thus) concentration.

In addition to employing light-induced cross-linking, our setup also allows samples to be ionically cross-linked *in situ* by filling the reservoir located in the bottom of the “hole-y plate” with, *e*.*g*., a calcium chloride (CaCl_2_) solution: then, the Ca^2+^ ions can diffuse through the membrane and enter the biopolymer solution. In the case of alginate samples, when using a CaCl_2_ concentration of 0.1 M in the reservoir, this process results in the formation of a viscoelastic gel with a final stiffness of around 6 kPa (see Supporting Information, Section S3 and Figure S2a), which indicates efficient ionic cross-linking of the alginate chains by the Ca^2+^ ions. For this particular ion/biopolymer combination, this result was expected and is typically described by the so-called “egg-box” model. However, for our porcine gastric mucin samples, a CaCl_2_ concentration of 50 mM (this concentration was reported to be optimal for the ionic cross-linking of bovine submaxillary mucins) has only a weak effect (see Supporting Information, Section S4) and creates a state that is just at the threshold toward a viscoelastic solid (= a gel).

In contrast to the covalent cross-linking strategy described above, ionic cross-linking comes with the advantage that it can, in principle, be reversed. This can either be achieved by diluting the ions by sample washing^39^ (which requires very long incubation times in a washing buffer of up to 24 h) or by adding a chelating agent that sequesters the ions and thus removes a previously formed cross-link.^44^ Here, we attempt to reverse the ionic cross-linking *in situ* by adding a chelator to the reservoir via the pumping system. Then, as the chelating agent diffuses through the membrane into the sample, it can gradually bind Ca^2+^ ions which results in a reduction of the viscoelastic moduli over time. However, when using only one chelating agent (*e*.*g*., either EDTA or citrate), we find only a slow/weak softening of the alginate gel, *i*.*e*., a reduction of the gel stiffness by about a factor of 3 after 1 h (see Supporting Information, Section S3 and Figure S2b). Thus, to achieve a stronger material response within the time scale of typical rheological measurements, we combine two chelating agents; and indeed, when using a mixture comprising 500 mM EDTA and 250 mM citrate, we observe a reduction of the stiffness of the Ca^2+^-cross-linked gel by 1 order of magnitude within ∼1 h (see Figure 2c).

To enable a third cross-linking mechanism, mucins were functionalized with thiolated DNA strands (crDNA, see Materials and Methods). Those crDNA strands are partially self- complementary and can act as a cross-linker by binding to other crDNA strands (see Figure 1) thus creating a mechanism that converts mucin solutions into gels.^45^ However, different from the ionic or covalent cross-links described above, those DNA-based cross-links form immediately when crDNA-functionalized mucins are brought into contact. Thus, time-dependent measurements are not very meaningful to demonstrate the implementation of this cross-linking strategy, which is why we compare frequency spectra obtained for unmodified mucins and crDNA-conjugated mucins to demonstrate successful gelation (Figure 2d). Moreover, such crDNA/crDNA cross-links can be removed again by the addition of a suitable displacement strand^45^ (dDNA, see Materials and Methods), which—in the example shown in Figure 2d—is efficient enough to turn a viscoelastic mucin gel back into a viscoelastic fluid.

Having explored different cross-linking options for the two pure biopolymer systems, we next study hybrid systems containing a 1:1 mixture of alginate and mucin. Our goal is to generate a well-mixed system of interpenetrating biopolymers, which can be dynamically cross-linked and (partially) unlinked on demand. To assess the miscibility of the two biopolymers, we produce a mixed sample in which a small fraction of either biopolymer component is fluorescently labeled (see Materials and Methods). Microscopy images of such an alginate/mucin blend demonstrate that, indeed, there is no visible phase separation between the two macromolecules (see Supporting Information Section S5 and Figure S4)—at least not at the length scale accessible to us by epifluorescence microscopy. This suggests that, when evaluating the macrorheological response of the mixed biopolymer system, we can expect both biopolymers to contribute to the viscoelastic properties and that cross-linking of either component should have a perceivable influence on the viscoelasticity of the mixture.

To test this expectation, we next measured the rheological response of such mixed networks upon activation/deactivation of cross-links *in situ*. We start with a combination of ionic and covalent cross-links, where the former are supposed to predominantly act on the (unmodified) alginate biopolymers whereas the latter should exclusively form between methyacrylated mucins/alginates.

In a first set of tests, 2% (w/v) mMUC is mixed with 2% (w/v) unmodified alginate, and the system is first covalently stabilized with UV light and then ionically cross-linked with 0.1 M CaCl_2_. With this particular order of the two cross-linking strategies, we observe gel formation upon illumination with UV light and obtain a gel stiffness of ∼30 Pa (see Figure 3a). When allowing Ca^2+^ ions to enter this covalently cross-linked network through the hole-y plate, the stiffness of the gel is further increased to ∼1.6 kPa. By exposing the double-cross-linked network to the chelator mix introduced above, the number of ionic cross-links can be successfully reduced, and the network is softened to ∼300 Pa after 11 min. Similarly, we also find a stepwise increase in the stiffness of the same biopolymer mixture when the order of the two cross-linking steps is switched: now, a gel stiffness of around 2.4 kPa is obtained after the first (= ionic) cross-linking step, and the second, covalent cross-linking procedure slightly increases this value to ∼ 2.6 kPa (Figure 3b, zoom in in Supporting Information, Section S3 and Figure S2c).

**Figure 3:**
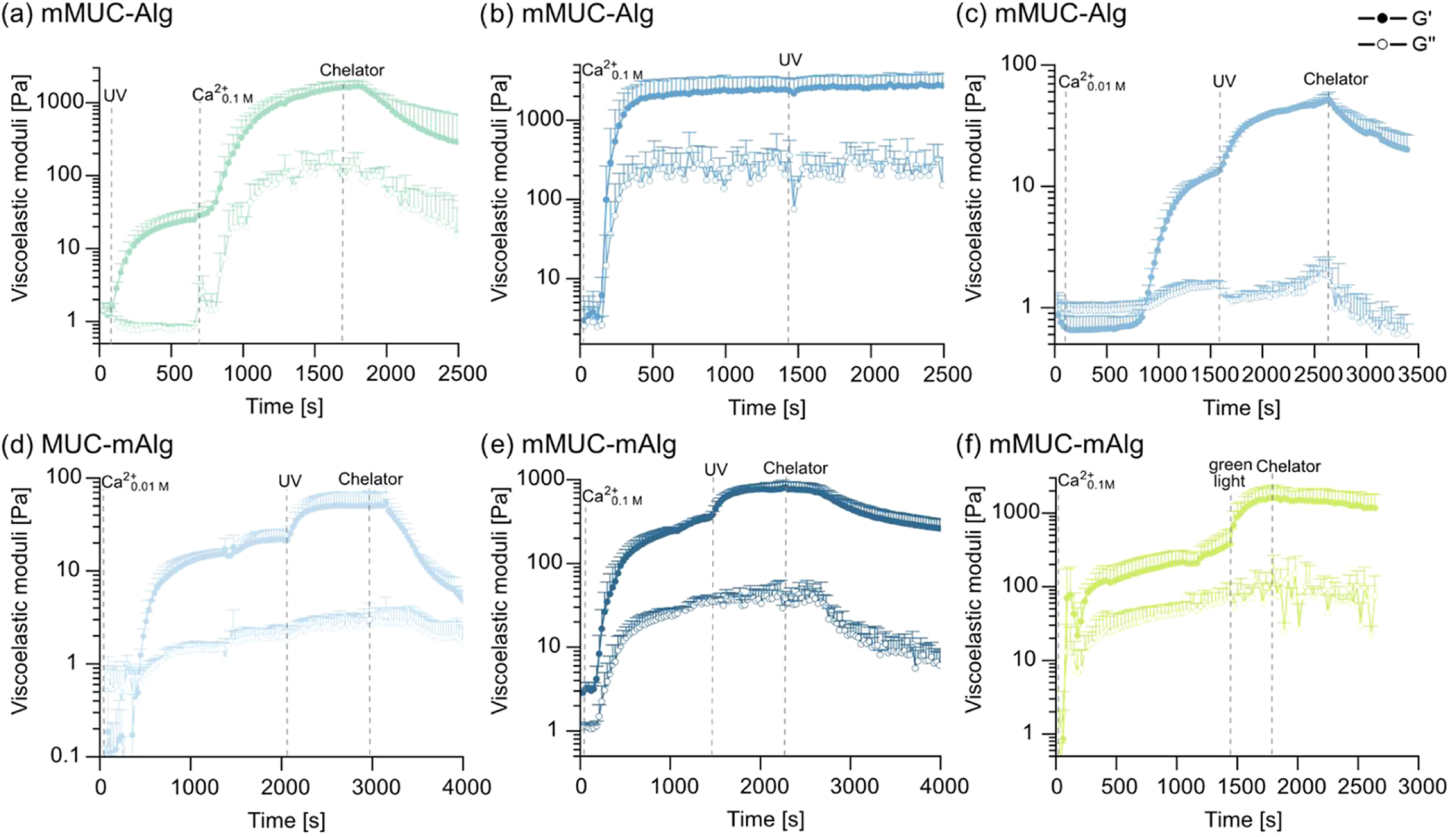
Viscoelastic properties of mixed alginate/mucin systems upon the activation/deactivation of cross-links *in situ*. In each sample 2% (w/v) of an alginate variant is combined with 2% (w/v) of a mucin variant; then the mixed sample is subjected to two consecutive cross-linking steps, after which the sample is exposed to a chelator mix. (a) An mMUC-Alg mixture is first cross-linked by illumination with UV light and then ionically cross-linked by adding 0.1 M CaCl_2_. (b) An mMUC-Alg mixture is first ionically cross-linked by adding 0.1 M CaCl_2_ and then covalently cross-linked using UV light. (c) An mMUC-Alg mixture is first ionically cross-linked by adding 0.01 M CaCl_2_ and then covalently cross-linked using UV light. (d) An MUC-mAlg mixture is first ionically cross-linked by adding 0.01 M CaCl_2_ and then covalently cross-linked using UV light. (e) An mMUC-mAlg mixture is first ionically cross-linked by adding 0.1 M CaCl_2_ and then covalently cross-linked using UV light. (f) An mMUC-mAlg mixture is first ionically cross-linked by adding 0.1 M CaCl_2_ and then covalently cross-linked using green light. Data shown represents mean values, error bars depict the standard deviation as calculated from *n* = 3 samples.

Of course, such a slight increase in stiffness (by 10% only) in response to the second cross-linking step is—for many applications—not sufficient; however, it is important to realize that the ionic cross-linking step has already created quite a stiff network in the range of a few kPa. We expect that, when starting with a lower baseline stiffness after ionic cross-linking, the additional covalent cross-links should have a stronger influence. Indeed, lowering the CaCl_2_ concentration by a factor 10 (to 0.01 M) has the desired effect: Now, we obtain a gel stiffness of ∼10 Pa after ionic cross-linking and ∼ 50 Pa after additional covalent cross-linking (Figure 3c).

When exposing this double-cross-linked network to the mix of chelating agents, we find a significant decrease in the gel stiffness after 15 min to ∼20 Pa (Figure 3c), which demonstrates the efficiency of this chelator mix.

Having demonstrated that it is possible to tune the mechanical properties of an alginate/mucin mixture in a (semireversible) two-step process, we next ask if a similar result can be obtained if both cross-linking agents target the same biopolymer component. In detail, we now mix 2% (w/v) unmodified mucin with 2% (w/v) mAlg and conduct the same cross-linking steps as in the previous example. As shown in Figure 3d, this adjusted strategy results in a similar outcome as described in Figure 3c, *i*.*e*., a switch from ∼20 Pa (obtained after ionic cross-linking) to ∼50 Pa, and back to ∼6 Pa after removing the ionic cross-links again. In other words, the activation and inactivation of the ionic cross-links both result in a change of the network stiffness by an order of magnitude. This suggests that, in this particular case, the chelators have fully removed the ionic cross-links from the network.

In several of those examples, dynamically switching the network mechanics up and down was very well possible—but on rather low levels of stiffness. Thus, in a next step, we ask if a similarly dynamic behavior can also be obtained for stiffer samples, which we aim to realize by mixing mMUC with mAlg. Now, different from the scenario described above (Figure 3b), the covalent cross-linking step should have a stronger influence on the system and thus should also result in a perceivable modulation of the network properties for samples with a higher initial stiffness (which are created by using the higher CaCl_2_ concentration of 0.1 M again). And indeed, as depicted in Figure 3e, this is the case: now, the network stiffness after ionic cross-linking is ∼400 Pa, after additional covalent cross-linking ∼800 Pa, and after chelator addition ∼400 Pa again (Figure 3e).

To test if a similar sequential activation of covalent and ionic cross-links is also feasible when employing green light, the same material combination of mMUC and mAlg is investigated again by changing the light source from UV to green light (Figure 3f). After bringing the system in contact with a 0.1 M solution of CaCl_2_, a hydrogel with a stiffness of ∼400 Pa is created due to ionic cross-linking, which agrees very well with the data shown in (Figure 3e). After activation of the covalent cross-links upon illumination with green light, however, the gel stiffness reaches values in the range of 1.5 kPa. As already observed for pure mAlg systems, also here, the application of green light seems to entail a stronger cross-linking of the sample compared to the result obtained with UV (see Figure 2c). After bringing the double-cross-linked sample into contact with the chelator solution, the stiffness is slightly decreased to ∼1.2 kPa. We speculate that, owing to the stronger effect of the covalent cross-linking obtained in this sample (compared to the conditions achieved with UV light), removing the ionic cross-links does not affect the sample stiffness as much as one might expect.

In other words, by relying on different ionic/covalent cross-linking steps in combination with differently functionalized biopolymer components, the mechanical properties of the same mixture of 2% (w/v) mucin and 2% (w/v) alginate can be dynamically switched back and forth on different levels of initial and final stiffness (see Figure 4). As shown in Supporting Information, Section S6 and Figure S5, with our approach, a dynamic switch of hydrogel stiffness is even possible several times in a row. This demonstrates that, when combining covalent and ionic cross-links, the addition and removal of Ca^2+^ ions can be repeated at will.

**Figure 4:**
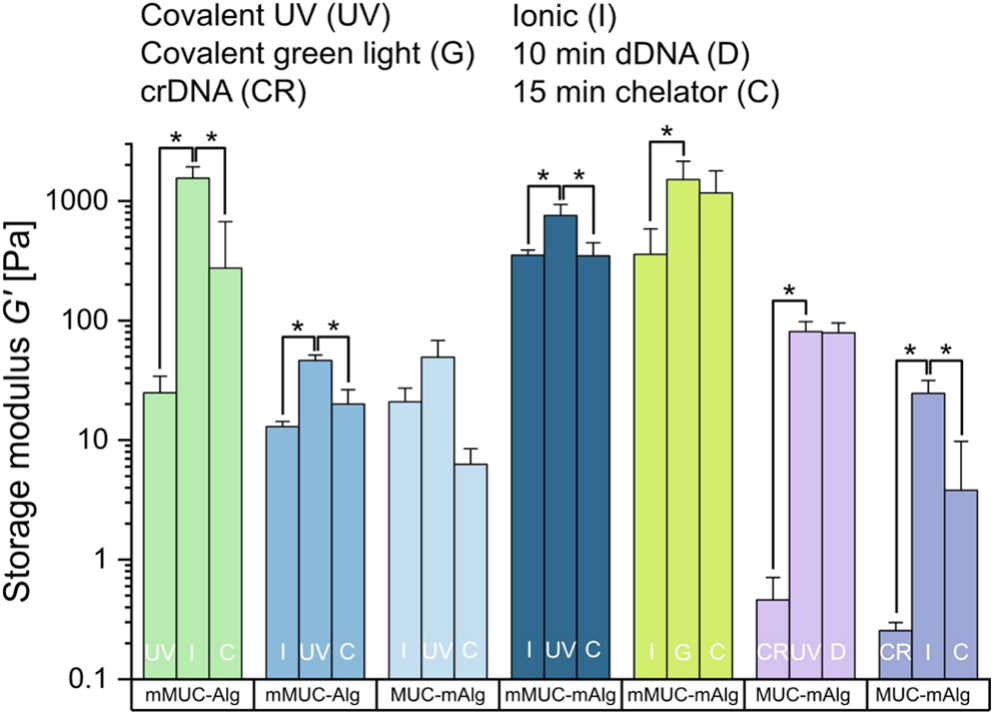
Overview over the storage moduli obtained for different mucin/alginate mixtures at different stages of cross-linker activation/inactivation. Always two different cross-linking strategies based on UV light (UV), green light (G), ionic cross-linking (I), or crDNA-assisted cross-linking (CR) are combined in a consecutive manner. Sample elasticities are shown after the first cross-linking step, after the second cross-linking step, and after (partial) cross-linker inactivation; the latter was achieved by either adding a chelating agent (C) or displacement DNA strands (D) to the samples *in situ*. Data shown represents mean values, error bars depict the standard deviation as calculated from *n* = 3 samples. Asterisks denote statistically significant differences based on a *p*-value of *p* = 0.05.

Of course, by varying the overall concentration of biopolymers and by adjusting the mixture ratio between alginate and mucin, even more options for tunability should be possible (see Supporting Information, Section S7 and Figure S6).

To better compare the effect of the different cross-linker activation/inactivation strategies described so far, a summarized overview of the storage moduli obtained at the different stages of hydrogel manipulation is shown in Figure 4. We note that, for many of the approaches tested here, a significant change in stiffness can be achieved via the stepwise application of the cross-linking/unlinking processes. Moreover, the distinct strategies allow us to reach different ranges of starting and final stiffnesses, thus rendering the composite material suitable for a variety of applications.

To this end, we have combined ionic and covalent cross-links in different ways; however, we have not employed DNA-based cross-links yet. In a previous study, we found that pure mucin systems stabilized by such DNA-based cross-links have a very low stiffness,^45^ and this result is confirmed here: for mixed networks comprising 2% (w/v) mucin and 2% (w/v) mAlg, crDNA-based cross-links entail gels with a stiffness of around 0.4 Pa (see Figure 5a). After exposing the sample to UV light, the stiffness of the gel is increased to ∼80 Pa; however, subsequent addition of dDNA strands (which are supposed to open the crDNA/crDNA cross-links) has no perceivable effect on the gel stiffness. This result is not surprising considering that the influence of the crDNA cross-links on the sample stiffness is very low—yet those crDNA-based cross-links are sufficient to provide a soft gel as a starting point for further sample modification.

**Figure 5:**
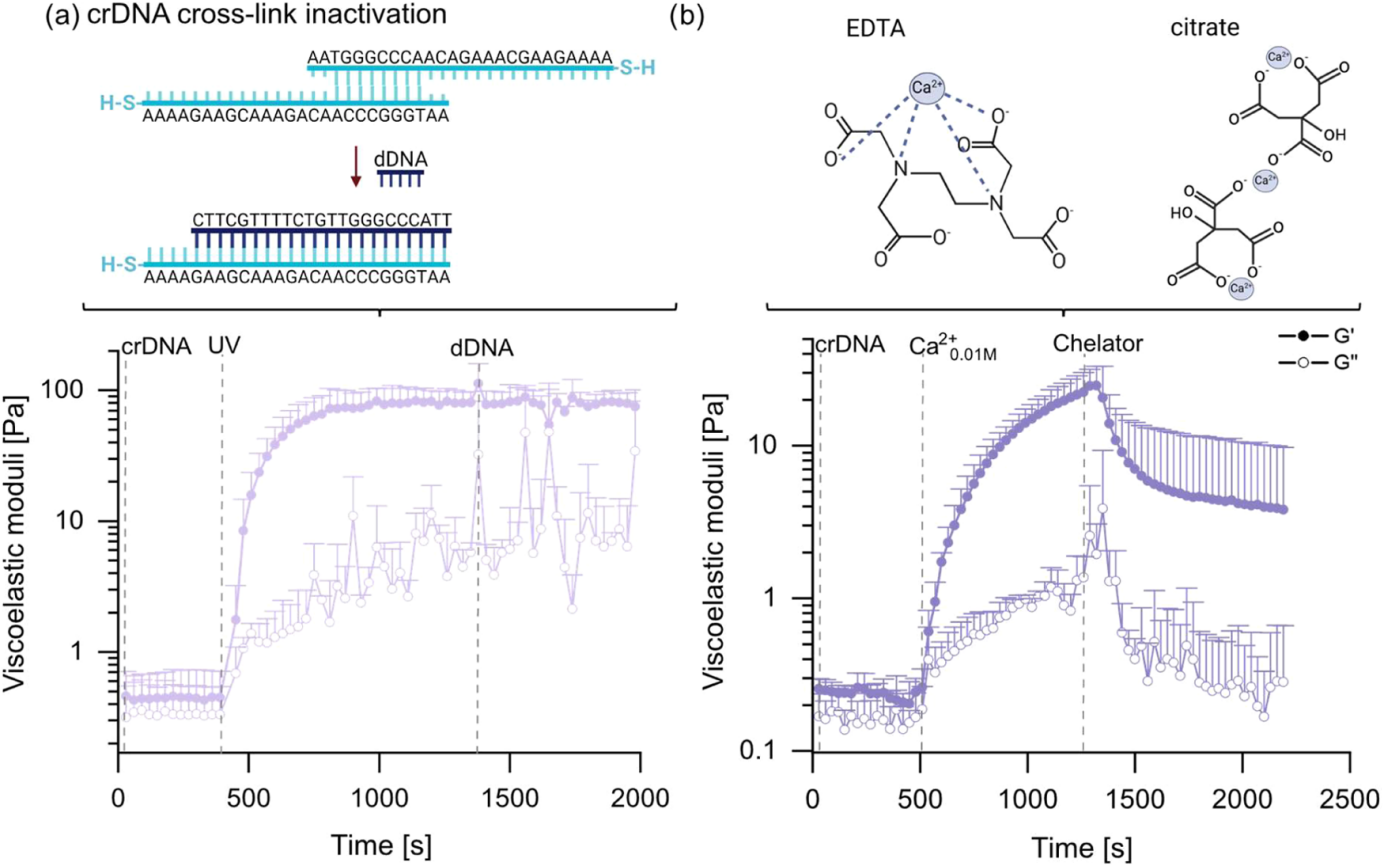
Viscoelastic properties of mixed alginate/mucin systems upon the activation/deactivation of cross-links *in situ*. A mixture comprising 2% (w/v) MUC and 2% (w/v) mAlg is treated with two consecutive cross-linking steps and one cross-linker deactivation step. (a) crDNA-based cross-links are combined with covalent cross-links (using UV light) and removing the DNA-based cross-link by adding dDNA. (b) crDNA-based cross-links are combined with ionic cross-links (generated by adding 0.01 M CaCl_2_), and the latter are removed by adding a chelator mix. The EDTA schematic shows an exemplary tetrahedral coordination; octahedral coordination is not depicted. Data shown represents mean values, error bars depict the standard deviation as calculated from *n* = 3 samples. The schematic drawings were made using BioRender: https://BioRender.com/s43b515.

Similarly, a soft mucin gel stabilized by crDNA cross-links can also be further stiffened by ionic cross-links: for instance, supplying 0.01 M CaCl_2_ (through the hole-y plate setup) to a soft mucin gel yields stiffness values of ∼25 Pa (see Figure 5b). Now, the ionic cross-links can be removed again by employing the same chelator mixture described above, which results in a reduced gel stiffness of around 4 Pa. Thus, with this third cross-linker variant based on DNA/DNA interactions, dynamically switching the stiffness of mixed mucin/alginate gels is also possible and extends the range of possible stiffness values toward the “soft” regime (see Figure 4).

The hydrogel system presented here comes with several options to vary the type and level of cross-linking, which should allow for tailoring the viscoelastic properties of the gels over a broad stiffness range. First, the overall biopolymer concentration (which was kept constant in this study for better comparability of the different systems) can be adjusted to soften or stiffen the hydrogels. As indicated in Supporting Information, Section S7, even without performing any cross-linking steps, such a strategy can already significantly affect the mechanical properties of the system. Second, in addition to varying the ion concentration used for cross-linking, also the type of cations used for ionic cross-linking can be altered. Here, trivalent ions would offer the option to bind to GG as well as GM groups (β-d-mannuronate (M) and α-l-guluronate (G) alginate subunits, which are arranged in polymannuronate (MM), polyguluronate (GG), and mixed GM domains) of alginate,^46^ which should result in a higher cross-linking density than what can be achieved with Ca^2+^. However, such an approach might affect the cytocompatibility of the system, which would require further investigations. Third, the degree of functionalization (here: methacrylation) of the employed biopolymers is a key parameter that can be adjusted to tune the effect of covalent cross-linking. In this context, also the concentration of the photoinitiator used can be varied to achieve a desired stiffness level. Thus, there are several parameters in the presented hydrogel system that can be tuned (more or less independently from each other) to further tailor the stiffness of the created material, either toward softer or stiffer hydrogels. In addition, altering the cross-linking strategy also affects the swelling and degradation behavior of the gels (see Supporting Information, Section S8 and Figure S7).

In a next step, to investigate the biocompatibility of the aforementioned systems for possible biomedical applications, we conduct cytotoxicity tests to determine the effect of the generated biopolymer gels themselves on the cell viability as well as the effect of the chemicals/light exposure used to generate the cross-linking processes. For doing so, human epithelial (HeLa) cells are incubated with medium which has been incubated with the different gel formulations prior to its application to the cells. This mimics a scenario where cells get into contact with the final, already cross-linked material (*e*.*g*., cell seeding tests), but are not directly exposed to the UV light or the cross-linking agents. Representative microscopy images of this experiment can be seen in Figure 6a. As depicted in Figure 6b, the MUC-mAlg blend system entails no significant decrease in cell viability compared to the control—and this outcome is independent of whether or not the photoinitiator required for cross-linking was added to the material (we find cell viability values of ∼ 93% in both cases). This suggests that the photoinitiator does not leave cytotoxic residues once the cross-linked system is created. In contrast, all the remaining blend systems investigated here lead to a significant decrease in cellular viability compared to the control group. However, all determined viability values are above 70%, and this is the threshold below which, according to ISO 10993–5, a material would be considered cytotoxic.

**Figure 6:**
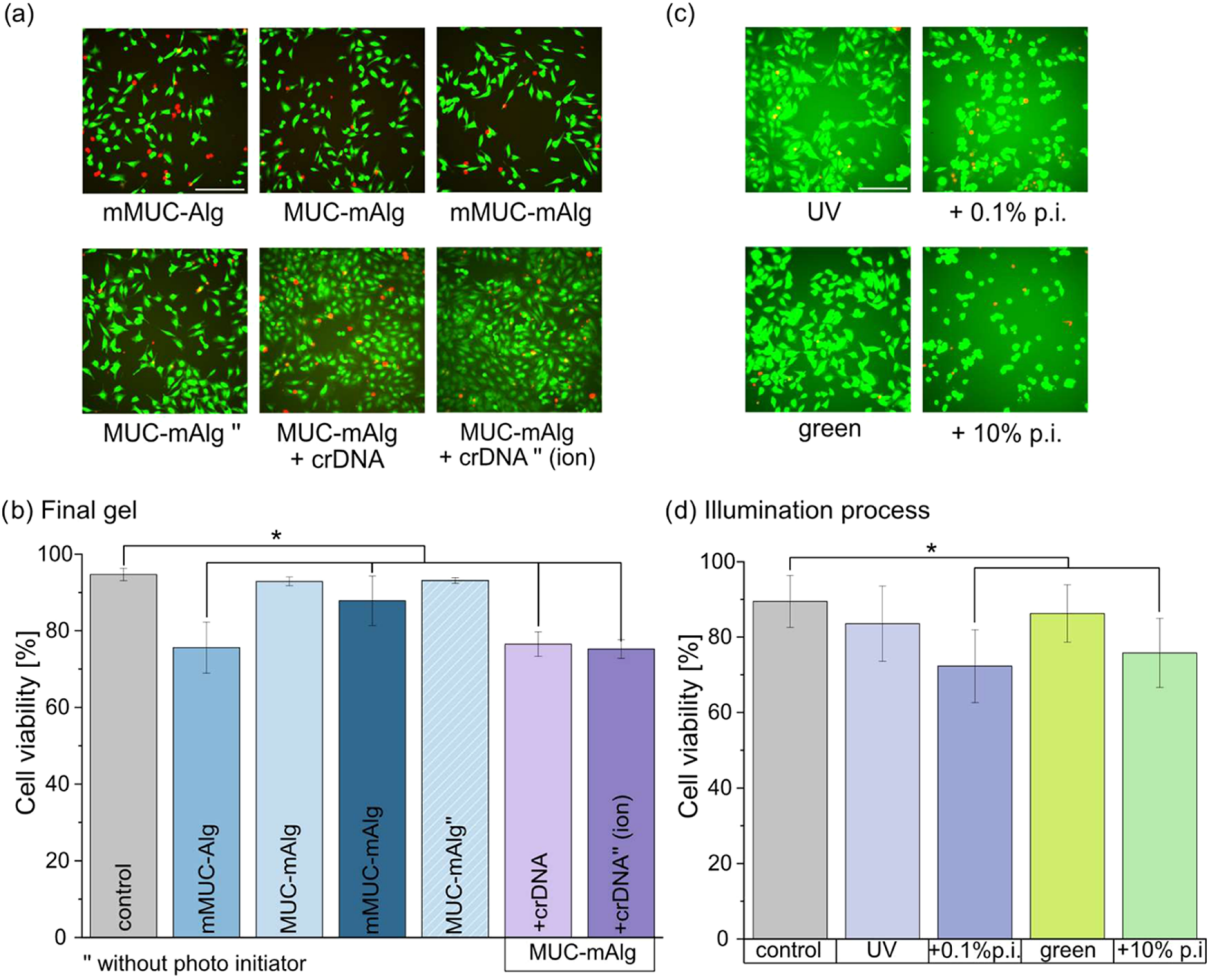
Viability experiments with HeLa cells to test for putative cytotoxic effects brought about by the hydrogel components or the chemicals/light exposure process used for cross-linking. (a) Representative images of cells stained with a live/dead staining solution. The images were obtained 1 h after the incubation process with the different materials was conducted. (b) Quantification of the images shown in (a) and similar ones. (c) Representative images of cells stained with a live/dead staining. The images were obtained 1 h after the cells were exposed to UV or green light (in the absence or presence of a suitable photoinitiator). (d) Quantification of the images shown in (c) and similar ones. Data shown represents mean values, error bars depict the standard deviation as calculated from *n* = 5 samples (from which 3 images were obtained each). Asterisks denote statistically significant differences based on a *p*-value of *p* = 0.05. The scale bars in the microscopy images represent 200 μm and apply to all images shown in this figure.

In addition to final gels created by cross-linking, the effect of two different light sources used during the cross-linking process and the photo initiators required for this procedure are investigated as well (for representative microscopy images see Figure 6c). Here, the cells are first covered with medium containing the corresponding photoinitiator and then exposed to one of the two different light types (*i*.*e*., UV or green light, see Figure 6d). The results obtained from these tests would be relevant for such applications, in which cells are directly embedded into the biopolymer mixture before gelation of the system is induced. Also here, we find cell viability values above 70% in all cases, which indicates the suitability of our systems for cell culture applications. In more detail, we determine a viability level of ∼84% for illumination with UV light and of ∼86% for illumination with green light, which is comparable to the control group. However, when conducting the light exposure tests in the presence of the respective photoinitiator, we find a significantly lower cell viability than for the control group, *i*.*e*., ∼72% (UV light) and ∼76% (green light). This indicates that the radical formation triggered by the photo initiators during either of the cross-linking reactions might impact the cell viability.^47,48^ In the cell viability tests summarized in Figure 6d, this issue did not occur since the cells were not in contact with the material when the cross-linking reaction employing light illumination was conducted. Together, this implies that, in those cases, the radical reaction was already completed when the cells were seeded onto the materials, or that only a low, unproblematic concentration of radicals was left at this time point. Still, when further tuning one of the mixed biopolymer systems for applications in *in vitro* cell culture assays or tissue engineering, it might be necessary to adapt the concentrations of the photo initiators and/or the light exposure times (see Supporting Information, Section S9). Data obtained after 24 h of incubation is shown in the Supporting Information (Section S9 and Figure S8), where it can be observed that only illumination with green light does not entail a significant decrease in viability compared to the control, and this underscores our expectation that UV light is more harmful to cells.

In a final step of this study, we aim at demonstrating the biological activity of the mucin component in the hybrid system by conducting incubation tests with bacteria. Those tests are motivated by previous findings, which showed that purified mucins can reduce the attachment of bacteria to coated surfaces and to bulk materials.^42,49,50^ Similar to those previous studies, here, the adhesion of different bacteria to mAlg/mMUC hydrogels is evaluated. In detail, we test both, Gram-positive (Staphylococcus aureus (S. aureus) and Streptococcus pyogenes (S. pyogenes)) as well as Gram-negative (Escherichia coli (E. coli) and Pseudomonas aeruginosa (P. aeruginosa)) bacteria and incubate them on mAlg samples containing different amounts of mucin (see Figure 7). As the alginate derived from seaweed used in this study has a similar biochemical structure as alginate produced by bacteria,^51^ we expect that pure mAlg gels do not possess strong antibacterial and antiadhesive properties themselves. And indeed, the ability of mAlg alone to reduce bacterial adhesion is low, especially for S. aureus and P. aeruginosa. For the other two bacterial strains, only a moderate effect of mAlg is observed. In contrast, pure mMUC samples significantly reduce the adhesion of all bacteria investigated here compared to pure mAlg samples, and this underscores the antibacterial effect of mucins reported in the literature.^42,49^ Here, the observed effect is weakest for P. aeruginosa, which agrees with previous findings investigating the bacteria-repellent effect of mucins toward this mucoadhesive bacterium.^42,52^

**Figure 7:**
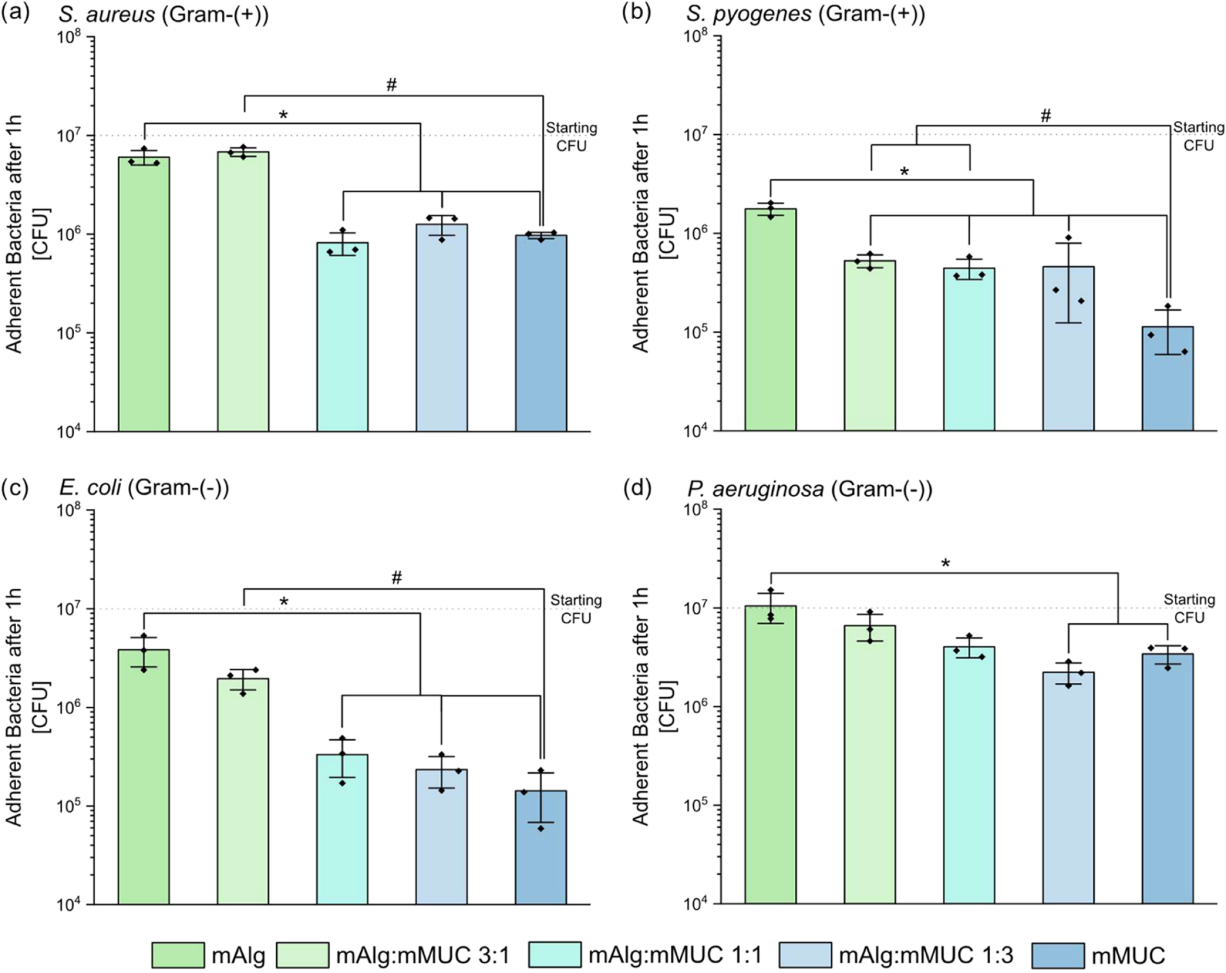
Incubation tests with different bacteria to evaluate the adhesion behavior of (a) S. aureus (Gram-(+)), (b) S. pyogenes (Gram-(+)), (c) E. coli (Gram-(−)), and (d) P. aeruginosa (Gram-(−)) to different hydrogel samples. 4% (w/v) mMUC samples, 4% (w/v) mAlg samples, and mixtures generated from those two biopolymers (at mixture ratios of 3:1, 1:1, and 3:1) with an overall biopolymer concentration of 4% (w/v) are compared. Data shown represents mean values, error bars depict the standard deviation as calculated from *n* = 3 samples. Significant differences compared to pure mAlg and pure mMUC samples are marked with an asterisk (*) and a pound symbol (#), respectively, and are based on a *p-*value of *p* = 0.05.

In full agreement with the results obtained for pure mAlg or pure mMUC systems, we find a dose-dependent reduction in the adhesion of the different bacteria when different concentrations of mMUC are integrated into the mAlg hydrogels. In most cases (except for S. pyogenes, see Figure 7b), a small mMUC content of 1% (w/v) (which corresponds to an mAlg/mMUC ratio of 3:1) does not entail a significant effect yet. However, at mixture ratios of mAlg and mMUC of 1:1 and above, a significant decrease in adhesion is observed for S. aureus (see Figure 7a), S. pyogenes (see Figure 7b) and E. coli (see Figure 7c). Only for P. aeruginosa (see Figure 7d), a mixture ratio of 1:3 is required to obtain a significant reduction in bacterial adhesion. If stronger antibacterial properties are required for certain applications, results reported by Zhang et al.^53^ suggest that incorporating certain concentrations of divalent ions, *i*.*e*., Ca^2+^ and Ba^2+^, into the hydrogel can boost its antibacterial properties. Of course, in this case, it would be necessary to check if and how the added ions affect the viscoelastic properties of the mixed system by inducing ionic cross-linking.

## Conclusions

In this study, we described different variants of well-mixed, biocompatible mucin/alginate composite hydrogels with dynamically switchable cross-links and antibacterial properties. Current hydrogel materials that allow for a dynamic switching of their viscoelastic properties mostly rely on synthetic polymers (*e*.*g*., polyacrylamide-azobenzene (PAMA) gels^54^ or hydrogels containing poly­(ethylene glycol) (PEG)^40,55^ ). There, especially the long time periods required to induce changes in the mechanical properties of those systems^39,40^ constitute a major drawback. Here, we obtained hydrogels, in which altering the viscoelastic response is rapidly possible. So far, we obtained the most promising results when combining ionic and covalent cross-links, or when combining DNA-based cross-links with ionic cross-links. The achievable gel stiffness range, within which such DNA-based cross-links are helpful tools to tune the mechanical properties of the hybrid system is, however, limited. Here, a modification of the DNA-based construct could be considered, *e*.*g*., to achieve higher cross-linking densities (and thus a stronger effect of the DNA-based cross-links on the gel stiffness) or to allow for “switching on” the DNA-based cross-links on demand. Previous work by Fujita et al.^56^ on hyaluronic acid based hydrogels cross-linked with single-stranded DNA suggests that this should be possible since, in that study, gels with stiffnesses of ∼100 Pa were obtained.

The ability to tailor the viscoelastic properties of the hybrid material in combination with the good biocompatibility of the biopolymer components renders this system a promising candidate for use in biomedical applications. By combining an advanced material design with real-time monitoring, our study contributes to the development of “switchable” hydrogels, which can have broad applications in microfluidics, tissue engineering, and regenerative medicine. For instance, making use of the dynamically switchable cross-links in mucin/alginate networks could be very interesting for biosensing, organ-on-chip technologies, or the microfluidic encapsulation of cells:^57^ in the context of the latter, the ability to change the mechanical properties of the cell environment on demand in combination with the antibacterial properties of the mucin might be very interesting to guide the differentiation of stem cells.^58^ Composite hydrogels with dynamic stiffness and the technical ability to monitor changes in their viscoelastic properties *in situ* could revolutionize these applications by enabling a precise control of their mechanical behavior and following the dynamics of the triggered alterations in real-time. Moreover, the hydrogel system proposed here could be a suitable model system for applications in the field of cellular research. In the case of certain diseases, a change of stiffness in the tissue surrounding the cells was observed.^59,60^ Here, being able to simulate such stiffness changes in a controlled manner to evaluate the ensuing response of cells embedded into the tunable hydrogel network can open new research avenues in this field.

## Materials and Methods

### Chemicals

Unless stated otherwise, all chemicals used in this study were purchased from Carl Roth GmbH & Co. KG (Karlsruhe, Germany).

### Mucin Purification

The mucin variant used in this study to generate hydrogels was derived from porcine gastric tissue and was manually purified to maintain the unglycosylated termini of the glycoprotein. Those termini are crucial for the project described here as they contain functional groups required for cross-linking. The mucin purification process was conducted according to a protocol described previously with slight changes.^61^ In brief, raw mucus was obtained by gently scraping the inner tissue surface of fresh, rinsed pig stomachs and subsequently diluted 5-fold in 10 mM PBS (pH = 7.0) containing 0.04% sodium azide and 170 mM sodium chloride (NaCl). This mixture was then stirred at 4 °C overnight, followed by filtration using a tea filter and an ultracentrifugation step (150,000 g at 4 °C for 1 h) to remove cellular debris. Afterward, size exclusion chromatography was employed to achieve a size separation of the mucins using an ÄKTA purifier system (GE Healthcare, Munich, Germany) with a XK50/100 column packed with Sepharose 6FF (GE Healthcare). Next, the mucin containing fractions were collected, the NaCl concentration was increased to 1 M, and the solution was dialyzed in double distilled water (ddH_2_O). Afterward, the mucin solution was concentrated by crossflow filtration (Xampler Ultrafiltration Cartridge, GE Healthcare; MWCO: 100 kDa) and finally lyophilized and stored until further use at −80 °C.

### Methacrylation of Mucin and Alginate

To obtain macromolecules capable of undergoing UV-triggered cross-linking, mucin (MW between 2 and 100 MDa,^62,63^ purified as described above) and sodium alginate (MW 342 kDa, see Supporting Information, Section S10, Merck, Darmstadt, Germany) were functionalized with methacryl groups. For doing so, 1% (w/v) of a given biopolymer was dissolved in ultrapure water. Then, this solution was cooled down to 4 °C while continuously stirring and the pH was adjusted to 8.0 using 1 M sodium hydroxide (NaOH). Per mg of biopolymer, 8.47 × 10^–4^ mL of methacrylic anhydride (Merck) were added to the solution and the pH was regularly readjusted to 8.0 for 24 h.^31,64^ Afterward, the sample was concentrated by centrifugation and dialyzed in ultrapure water for 3 days. The concentrated biopolymer solutions were subsequently lyophilized and stored at – 80 °C until further use.

### ATTO Labeling

MUC and mAlg were labeled with ATTO 594 (*E*_x_/*E*_m_: 603:636 nm) and amine modified ATTO 425 (*E*_x_/*E*_m_: 439:585 nm, ATTO-TEC GmbH, Siegen, Germany), respectively, using 1-ethyl-3-(3-(dimethylamino)­propyl) carbodiimide hydrochloride (EDC, Carl Roth) and *N*-hydroxysulfosuccinimide sodium salt (NHS, 98% purity, abcr GmbH, Karlsruhe, Germany) for carbodiimide chemistry. For imaging such labeled macromolecules, samples were prepared with a total macromolecular content of 4% (w/v). Here, from each biopolymer component, only a small fraction (5% (w/v) of the respective component) was carrying a fluorescent label. The mixed systems were then imaged on fluorescence microscope (DMi8, Leica, Wetzlar, Germany).

### DNA Design

To generate DNA-cross-linked hydrogels and to achieve a controlled opening of such DNA-based cross-links, DNA sequences designed by Nowald et al.^65^ were used. Here, a single-stranded DNA (crDNA) sequence comprising 26 bases acts as a cross-linker: those strands can partially bind to themselves as they exhibit a self-complementary subsequence of 8 bases in length. Fully complementary dDNA strands comprising 22 bases are designed such that they have a higher binding affinity to the crDNA strands than the crDNA strands have to themselves. Thus, when dDNA strands are added to a hybridized crDNA/crDNA pair, they will open this hybridization by triggering a displacement reaction. Previously, the online DNA analysis tool NUPACK has been used to confirm the high probability of forming the envisioned base pairings described above; moreover, it was shown that these DNA sequences do not form any secondary structures in the presence of 5 mM Mg^2+^ and 150 mM Na^+^ at 37 °C.^45,66^ As in this previous study, thiol-modified crDNA was used here to attach the crDNA strands to the cysteines of mucin.

All synthetic DNA strands were purchased from Integrated DNA Technologies (Munich, Germany). The different oligonucleotide sequences used for hydrogel cross-linking and cross-link opening are listed in Table 1.

**Table 1:** Oligonucleotide Sequences Used in This Study

strand type	sequence (from 5′ to 3′)	modification (at the 5′ end)
cross-linker DNA (crDNA)	AAA AGA AGC AAA GAC AAC CCG GGT AA	5ThioMC6-D
displacement DNA (dDNA)	TTA CCC GGG TTG TCT TTG CTT C	

### Spectroscopy

To assess the efficacy of the methacrylation process, light absorption measurements were performed. Therefore, the methacrylated biopolymers were each dissolved in ultrapure water, and their light absorption behavior was characterized at wavelengths between 200 and 260 nm. To determine the achieved degree of methacrylation, the obtained absorption values at 220 nm were compared to those of a dilution series of a pure methacrylic anhydride solution (for a standard curve, see Supporting Information, Section S11). By doing so, the number of methacrylic anhydride groups per molecule was determined to be around 26 for MUC and 18 for Alg. For crDNA, a cross-linking efficiency of ∼8% was determined (see Supporting Information, Section S12).

### Sample Preparation

To obtain hydrogels with different viscoelastic properties, the biopolymers alginate, mucin, methacrylated alginate, methacrylated mucin, and DNA-conjugated mucin were reconstituted into aqueous solutions and cross-linked gels at different formulations. To obtain cross-links between distinct biopolymers, a combination of different mechanisms (see Figure 1) was considered.

#### Ionic Cross-Linking of Alginate

Ionically cross-linked hydrogels were prepared by dissolving sodium alginate in ultrapure water and adding a 0.1 or 0.01 M CaCl_2_ solution into the reservoir of the “hole-y plate”. A combination of chelators was used to partially remove these ionic cross-links generated by Ca^2+^, *i*.*e*., (see Supporting Information, Section S3). The addition of the ion and chelator solutions, respectively, was performed *in situ* and is described in more detail in the section ‘rheological setup for *in situ* measurements’.

#### Covalent Cross-Linking by Illumination with UV Light

To generate covalently cross-linked hydrogels using UV light, methacrylated alginate or mucin was dissolved in ultrapure water. In parallel, the photoinitiator 2-hydroxy-4′-(2-hydroxyethoxy)-2-methylpropiophenone (Merck) was dissolved at a concentration of 10% (w/v) in 80% ethanol; from this stock solution, 10 μL were later added per 1 mL of biopolymer solution.^67^ Then, the biopolymers were cross-linked by exposing them to UV light (M365L2; wavelength: 365 nm; output power: 190 mW; ThorLabs, New Jersey) for several minutes.

#### Covalent Cross-Linking by Illumination with Green Light

To generate covalently cross-linked hydrogels using a green light source (5 m LED, 532 nm, 3200 lm/m, Novectro GmbH & Co KG, Hörbranz, Austria), samples were prepared at a final concentration of 2% (w/v) mAlg and 2% (w/v) mMUC for hole-y plate measurements and at 2% (w/v) mMUC or 4% (w/v) mAlg for other rheological experiments. Then, 10% (v/v) of the cross-linking solution dissolved in ddH_2_O was added to those solutions, which resulted in final concentrations of 1 mM eosin-y, 125 mM triethanolamine (TEOA), and 20 mM 1-vinyl-2-pyrrolidone (VP, Merck). Here, eosin-y is excited when illuminated with green light, and then reacts with TEOA by forming radicals that initiate the cross-linking process.^68,69^ VP acts as a catalyst accelerating the reaction.^70^

#### Preparation of DNA-Cross-Linked Mucin Hydrogels

To obtain a cross-linked hydrogel of mixed mucin/alginate components, the previously published protocol for generating cross-linked mucin hydrogels based on crDNA was adjusted.^45^ Overall, the cysteines of mucins were targeted to form disulfide bonds with crDNA strands. Therefore, crDNA was dissolved in RNase-free water containing 0.1 mM EDTA (Thermo Fisher Scientific, Waltham) to a final concentration of 30 μM and 3 mM tris­(2-carboxyethyl)­phosphine-hydrochloride (TCEP-HCl); then the sample was incubated at room temperature for 2 h while shaking slightly. The purpose of this step is to enable access to the thiol groups by reducing the protective S - S bonds located at the termini of the DNA strands. In a next step, 3% (w/v) of mucin dissolved in ddH_2_O was mixed with the crDNA solution to a final mucin concentration of 2% (w/v), and the sample was stored at 4 °C overnight. In the case of mixed systems, a solution with a concentration of 2% (w/v) MUC and 2% (w/v) alginate was prepared and crDNA was added to a final concentration of 30 μM.

With the crDNA strands, a direct formation of cross-links via the self-complementary nature of those DNA strands is possible. Then, the addition of dDNA strands with a larger number of complementary base pairs toward crCNA should entail an opening of the crDNA/crDNA cross-link. For that purpose, an excessive amount of dDNA (50 μM) was added to the cross-linked mucin gel. The interaction of these different DNA strands is illustrated in Figure 5. Each experiment was performed with *n* = 3.

### Rheological Setup for *In Situ* Measurements

#### “Hole-y Plate” Setup

The setup illustrated in Figure 8 allows for the addition of cross-linking or cross-linker modifying agents *in situ*. This setup comprises a custom-made bottom plate designed for use in Anton Paar rheometers and enables the diffusive entry of molecules from a fluid reservoir through a membrane into the sample while simultaneously avoiding the sample to leak into the fluid reservoir.

**Figure 8:**
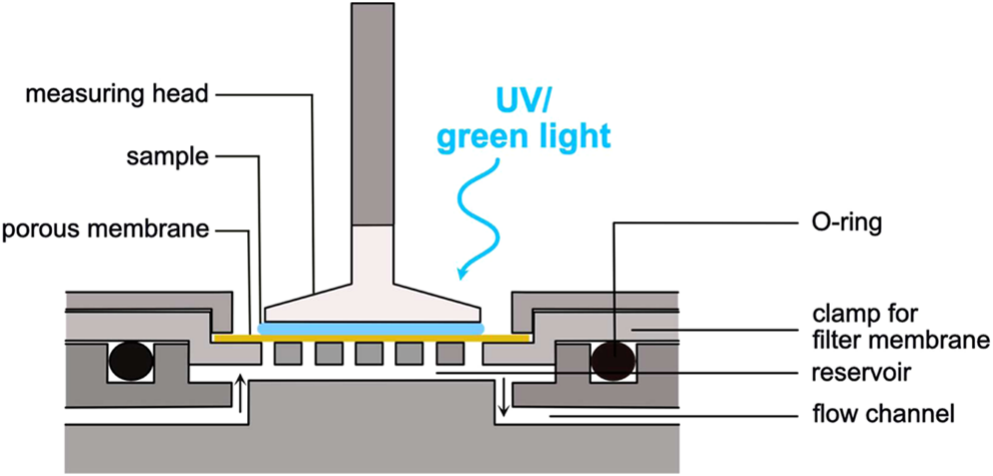
Schematic representation of the “hole-y plate” setup used in this study.

The membrane used for this application is made from poly­(vinylidene fluoride) (PVDF; Microlab Scientific Co., Ltd., Shanghai, China), has a pore size of 0.22 μm and hydrophilic properties. In a pretest, we verified that a diffusive translocation of the synthetic dDNA strands used here across this membrane is indeed possible (see Supporting Information, Section S13). To obtain reproducible measurements with this setup, it is key to ensure a proper fitting of the membrane piece: A too large membrane becomes wavy and touches the measuring head, thus interfering with the measurement. However, if the cut membrane piece is too small, one of two artifacts can occur: first, sample material from the measuring chamber can enter the reservoir located in underneath, get cross-linked and thus prevents the diffusive entry of Ca^2+^ ions into the sample; second, larger amounts of the cross-linking solution can enter the sample chamber thus resulting in stronger cross-linking of the sample than desired.

The fluid reservoir has a volume of around 400 μL. During a measurement, the fluid in this reservoir can be exchanged via an attached syringe pump system, which is connected to the hole-y plate via a tube (diameter of ∼1 mm) using needles (diameter of 0.8 mm, B. Braun, Melsungen, Germany) and a Luer-lock adapter. When processing ion solutions or chelating agents, the pump was set to a throughput rate of 0.5 mL/min. When processing DNA solutions, the settings were adapted to 50 μL/min for dDNA and 200 μL/min for CaCl_2_ and the chelator solution. For very soft samples, switching on the pump during a running measurement induced artifacts in the recorded data (see Supporting Information, Section S14), which were then removed before averaging curves. Each experiment was performed with *n* = 3.

#### Setups for Light-Triggered Cross-Linking

In this study, two different setups for the light-induced cross-linking of biopolymers *in situ* were employed: A commercially available, UV-transparent rheometer plate (P-PTD200/GL, Anton Paar, Graz, Austria) was used for sample illumination from below. However, this measuring setup does not allow for the dynamic addition of molecules to the sample during a measurement as it prevents usage of the “hole-y plate” described above. Thus, to enable a light-triggered cross-linking process from above (that is compatible with the use of the ‘hole-y plate’), a bespoke measuring head was developed, that allows for the transmission of visible/UV light. This measuring head (see Supporting Information, Section S2) can be connected to a commercial adapter (D-CP/PP7, Anton Paar) and was fabricated from a certain poly­(methyl methacrylate) variant (PMMA, Plexiglas XT 0A070, Röhm GmbH, Darmstadt, Germany), which has a low absorbance behavior in the UV range (see Supporting Information, Section S2).

### Shear Rheology Tests

The viscoelastic properties of the samples were determined using a research-grade shear rheometer (MCR302, Anton Paar) equipped with a planar measuring head PP 25 (PP25/Q1–79044, Anton Paar). Depending on the setup, either a temperature-controllable planar bottom plate (P-PTD200/56, Anton Paar) or a plate with a thread (P-PTD200/80/1, Anton Paar) to attach the hole-y plate was used. Before performing any measurement, the setup was initialized and calibrated. When using the hole-y plate, the gap size for the measurements was set to 0.4 mm; when analyzing DNA containing samples, the gap size was reduced to 0.1 mm to reduce the required sample. Sample volumes of 200 and 100 μL, respectively, were carefully placed onto the measuring plate before lowering the measuring head into its position. A gelation curve and/or frequency spectra (obtained for oscillation frequencies of *f* = 0.1 to 10 Hz) were conducted after 60 s of equilibration time; in either case, the storage and the loss modulus of the sample were recorded. A pre-experiment was carried out before every frequency sweep to guarantee linear material response; here, the shear strain was set to 1.5 times the average shear strain obtained in 5 repetitions at an oscillatory torque of 0.5 μN·m. All rheological experiments were conducted at a constant temperature of 20 °C. Each experiment was performed with *n* = 3.

#### Chelator Induced Removal of Ionic Cross-Links

After ionically cross-linking alginate-based hydrogels, the cross-links were partially removed by binding the incorporated ions with chelators. For doing so in an *in situ* process, the reservoir was first flushed with ddH_2_O to completely remove the cross-linking agent from the hole-y plate. Afterward, another syringe was connected to the setup and filled with the chelating agent, *i*.*e*., a mixture of 0.5 M EDTA (powder) and 0.25 M citrate solution.

#### DNA Controlled Opening of Cross-Links

To open the DNA-based cross-links in mixed mucin/alginate hydrogels, dDNA was added to the sample. Therefore, a solution of 50 μM dDNA prepared in RNase-free water containing 0.1 mM EDTA was added to the sample via the hole-y plate setup.

Here, the aforementioned pumping system was used to fill the reservoir of the hole-y plate with the dDNA solution. To better detect the weak effect that DNA-based cross-links have on the sample stiffness, the rheometer settings for obtaining the gelation curves were adapted to 0.1 μN·m and 0.2 Hz to achieve a higher sensitivity.

### Cytotoxicity Tests

To investigate the effect of the different material combinations as well as the cross-linking agents on the viability of cells, a cytotoxicity assay was performed with HeLa cells. Here, 2 mL of the different materials (mMUC-Alg, MUC-mALg and mMUC-mAlg) were prepared and cross-linked with UV light, crDNA, and/or 0.01 M CaCl_2_. Afterward, 3 mL of cell culture medium (minimum essential medium eagle (Merck) supplemented with 10% (v/v) fetal bovine serum (Merck), 1% (v/v) l-glutamine solution (Merck), 1% (v/v) penicillin-streptomycin (Merck), and 1% (v/v) nonessential amino acids (Merck)) was added to each sample and incubated at 37 °C and 5% CO_2_ overnight. HeLa cells were seeded into a 96 well plate (10,000 cells per well, Nest Biotechnology Co. Ltd., Wuxi, China) and incubated overnight at 37 °C and 5% CO_2_. The next day, the incubated cell culture medium was sterile filtrated using 0.22 μm filters to achieve sterile conditions, and 100 μL of the obtained solution was added to each well; the cells were incubated with this medium at 37 °C and 5% CO_2_ overnight. Afterward, the cells were stained with a live/dead staining comprising 1 μM calcein and 2 μM ethidium homodimer-1 (Invitrogen, Carlsbad, CA) and subsequently imaged using a fluorescence microscope (Leica) with a 20× objective (HC PL FLUOTAR L 20×/0.40 DRY) and a digital camera (Orca Flash 4.0 C11440, Hamamatsu, Japan) using FITC (*E*_x_ = 460–500, DC = 505; *E*_m_ = 512–542, Leica) and TXR filter cubes (*E*_x_ = 540–580, DC = 585; *E*_m_ = 592–668, Leica). The such recorded images were evaluated using the software ImageJ (version 1.54g). As a negative control, the cells were incubated with sterile methanol for 2 min and washed with Dulbecco’s phosphate buffered saline (DPBS, Merck) before further processing. Imaging results demonstrate that this method kills all cells (data not shown).

The effect of the light exposure process required for covalent cross-linking (using UV or green light) was assessed using HeLa cells as well. Therefore, HeLa cells were seeded into 96 well plates with a glass bottom (10,000 cells per well, Greiner Sensoplate, Greiner Bio-One, Kremsmünster, Austria) to allow for light exposure from below. The seeded cells were incubated in 100 μL of cell culture medium overnight at 37 °C and 5% CO_2_. For the viability assessment on the next day, the culture medium was replaced with 150 μL of either pure medium or medium supplemented with a photoinitiator. The well plate was subsequently illuminated with either UV light (for 10 min) or with green light (for 5 min). Afterward, the wells were washed with DPBS (Merck) and stained with the aforementioned live/dead staining solution. Subsequently, the samples were imaged using a fluorescence microscope (Leica) and analyzed with the software ImageJ. The cells were then incubated overnight, and the staining and imaging process was repeated the next day. Cells incubated with sterile methanol for 2 min and washed with DPBS (Merck) before further processing were again used as a control group; also here, all cells were killed by the methanol exposure (data not shown). Each experiment was performed with *n* = 5, from which 3 images were obtained each.

### Biofouling Tests with Bacteria

Samples comprising mMUC, mAlg, as well as their mixtures (prepared at ratios of 1:1, 1:3 and 3:1) were prepared at an overall biopolymer concentration of 4% (w/v); each sample contained 0.1% (w/v) of photoinitiator. The hydrogel samples were prepared in 500 μL reaction tubes with a standardized surface area, cross-linked, and sterilized by illumination with UV light in a sterilization chamber (BLX-254, Vilber Lourmat GmbH, Eberhardzell, Germany; UV light: 254 nm, 5 × 8 W) for 30 min.

Bacterial adhesion to the different hydrogel samples was determined using a modified version of a previously published protocol.^71^ All attachment tests were conducted with the following bacterial strains: S. aureus USA300 Lac (JE2), S. pyogenes ATCC 700294, E. coli 536, and P. aeruginosa PAO1 (see Supporting Information, Section S15 for further information). Each strain was cultured to reach 10^8^ colony forming units (CFU) per mL in the appropriate medium. To each hydrogel sample, 100 μL of the bacterial suspension (10^7^ CFUs) were added, and the samples were incubated at 37 °C for 1 h without shaking. Following incubation, nonadherent bacteria were removed by washing the samples with sterile PBS (3 × 100 μL), followed by decanting. To detach the remaining adherent bacteria, the samples were vortexed in 100 μL of fresh PBS for 1 min or until the hydrogel dissolved. The resulting suspensions were serially diluted (10^–3^ to 10^–6^, depending on the strain and conditions) and plated onto 1.5% agar plates containing the respective growth medium. The plates were incubated at 37 °C for 24 h (or for 48 h in the case of S. pyogenes). After this incubation step, bacterial colonies were counted to determine the number of adherent bacteria on each hydrogel surface. All conditions were tested in three biological replicates, with each consisting of three technical replicates.

### Statistics

Statistical tests were conducted using the software OriginLab (Northampton, Massachusetts). In a first step, a Shapiro-Wilk test was performed to test for normal data distribution. Subsequently, a two sample Student’s *t* test (in the case of Figure 4, a one-sided *t* test) was conducted to normally distributed populations with similar variances whereas a Wilcoxon signed-rank test was performed in situations where the variances were unequal. In case the values were not normally distributed, a Mann–Whitney test was employed. A *p*-value of *p* ≤ 0.05 (corresponding to a confidence level of 95%) was selected as a threshold for significance; in the figures, significant differences were labeled with an asterisk or a hashtag.

## Supplementary Material



## Data Availability

The data sets generated during this study are available from the corresponding author upon reasonable request.

## Notes

Comparison of different polymers; UV-transparent measuring head for rheology; chelating agents; ionic cross-linking of mucin; homogeneity of mixed alginate/mucin samples; dynamic change of stiffness in cycles; degradation/swelling study; influence of the mixing ratio on the viscoelastic moduli of un-cross-linked mMUC/Alg samples; effect of green light on cell viability; molecular weight determination of alginate; degree of methacrylation; conjugation efficiency of mucin with crDNA; permeability of the filter membrane toward DNA strands; artifacts created by the pump during measurements with crDNA; bacterial strains and media (PDF)
