# Supplementary material for: Reversible Stiffening of Biopolymeric Hybrid Networks by Dynamically Switching Cross-Links In Situ
Source: ACS Appl Mater Interfaces. 2025 May 13;17(20):29276–90. doi: 10.1021/acsami.5c03419 (PMC12100592; doi:10.1021/acsami.5c03419)
Supplement: Supplementary file 1 [file am5c03419_si_001.pdf]

Supporting Information for

Reversible Stiffening of Biopolymeric Hybrid Networks by Dynamically Switching Crosslinks *in situ*

Jana T. Reh[1,2,3], Sebastian Voigt[1,2,3], Leonard R. Gareis[3,4], Ufuk Gürer[1,2,3], Stephan A. Sieber[3,4], Berna Özkale[3,5,6], Oliver Lieleg[1,2,3]*

[1] School of Engineering and Design, Department of Materials Engineering, Technical University of Munich, Boltzmannstraße 15, 85748 Garching, Germany

[2] Center for Protein Assemblies (CPA), Technical University of Munich, Ernst-Otto-Fischer Straße 8, 85748, Garching, Germany

[3] Munich Institute of Biomedical Engineering, Technical University of Munich, Boltzmannstraße 11, 85748 Garching, Germany

[4] TUM School of Natural Sciences, Department of Bioscience, Chair of Organic Chemistry II, Technical University of Munich (TUM), Ernst-Otto-Fischer-Str. 8, Garching 85748, Germany

[5] Microrobotic Bioengineering Lab (MRBL), Department of Electrical Engineering, School of Computation, Information and Technology, Technical University of Munich, Hans-Piloty-Straße 1, 85748, Garching, Germany

[6] Munich Institute of Robotics and Machine Intelligence, Technical University of Munich, Georg-Brauchle-Ring 60, 80992 Munich, Germany.

*: corresponding author

Center for Protein Assemblies (CPA), Technical University of Munich,

Ernst-Otto-Fischer Str. 8, 85748 Garching, Germany

e-mail: oliver.lieleg@tum.de

keywords: reversible crosslinks, biopolymer, tunable stiffness, hydrogels, anti-bacterial

ORCID: **JTR** (0009-0005-1462-8311); **SV** (0009-0005-4999-0177); **LRG** (0009-0004-2278-7200); **UG** (0009-0004-0016-7406); **SAS** (0000-0002-9400-906X); **BÖ** (0000-0002-3016-9363); **OL** (0000-0002-6874-7456)

Section S1: Comparison of different biopolymers suitable for hydrogel applications

The following Table S1 gives an overview of different biopolymers commonly used for creating hydrogels with applications in biomedical research and lists their characteristics, the complexity of the corresponding hydrogel preparation process, and their costs.

Table S1. Comparison of different biopolymers suitable for hydrogel generation according to their characteristics, hydrogel preparation complexity, and prize. Cost* was compared on the basis of prices listed on sigmaaldrich.com (May 2025), with costs per 100 g being: €: up to 70 €; €€: 70 – 200 €; €€€: above 200 €. (RT: room temperature).

Polymer	Characteristics	Preparation Complexity	Cost*
Alginate[1]	• biocompatible<br>• functional groups for modifications	• dissolution in water at RT<br>• easily cross-linkable	€
MUC5AC[2]	• biocompatible<br>• anti-biofouling<br>• anti-viral/anti-inflammatory<br>• many functional groups for modifications	• dissolution in water at RT<br>• easily cross-linkable when modified	€€€ (lab-purified)
Chitosan[3]	• biocompatible<br>• anti-bacterial<br>• forms gels under acidic conditions	• dissolution in acidic conditions required	€€
Gelatin[4,5]	• biocompatible<br>• thermosensitive<br>• batch-to-batch variation	• dissolution in water above ~35°C	€
Hyaluronic acid[6]	• biodegradable<br>• promotes wound healing<br>• suitable for soft hydrogels	• dissolution in water at RT	€€€
Cellulose[7,8]	• biocompatible<br>• good mechanical and chemical stability	• limited solubility in water	€
Fibrin[9]	• bioactive<br>• interaction with cells<br>• quick degradation<br>• poor mechanical properties	• requires a combination of fibrinogen and thrombin	€€€

Section S2: UV-transparent measuring head for rheology

To be able to expose samples to UV light *in situ* and from above, a dedicated measuring head was fabricated from polymethyl methacrylate (PMMA). Figure S1a depicts a schematic drawing of this bespoke measuring head. Its upper section was designed to fit into a commercial adapter (D-CP/PP7, Anton Paar) of the rheometer whereas its lower section comprises a flat, round surface with a diameter of 25 mm.

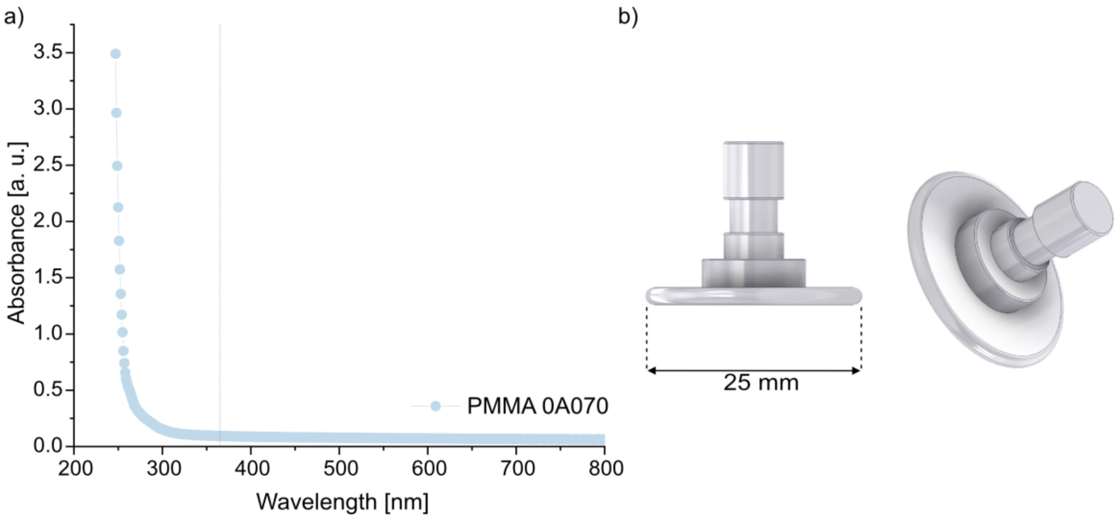

Figure S1. Absorbance spectrum of the PMMA variant used here for crafting the bespoke measuring head (*n* = 1) (a) and schematic drawing of the UV-transparent measuring head developed for this project (b).

As shown in Figure S1b, the PMMA variant used for this custom-made measuring head has low absorbance values at 365 nm and is thus suitable for the UV-crosslinking of samples *in situ*. The absorbance spectrum was determined on a UV-VIS spectrometer (Varioskan LUX multimode microplate reader, Thermo Fisher Scientific, Waltham, USA).

Section S3: Chelating agents

In this study, we not only aim at creating ionic cross-links *in situ* (see Figure S2a), we also aim at reducing the cross-link density again by adding chelating agents to the samples. Here, to avoid acidic gelation of the mucin component in the samples, the pH values of the chelator solutions as well as the ion solution were measured and all found to be either in a neutral or basic pH range (see Table S2).

Table S2. pH values of the chelator solutions and $CaCl_2$ solution used in this study.

solution	pH value
0.5 M citrate	7.1
0.5 M EDTA	8.9
0.5 M EDTA + 0.25 M citrate	11.9
0.1 M $CaCl_2$	7.9

This set of chelator solutions was examined to find a chelating agent that efficiently removes the ionic cross-links from pure alginate samples. Among the options tested, a combination of 0.5 M EDTA with 0.25 M citrate resulted in the strongest decrease of the viscoelastic moduli (see Figure S2b); thus, this combination was chosen for further experiments. A higher EDTA concentration was not tested further as we observed corrosive damage to the measuring head in pilot experiments. The 0.5 M citrate solution used in the experiments was obtained by dissolving 12.1345 g of sodium citrate dihydrate and 1.679 g of citric acid (VWR, Leuven, Belgium) in 100 mL of ddH_2O.

When investigating the composite mMUC-Alg hydrogel, the UV cross-linking step following the ionic cross-linking with Ca^{2+} did not result in a strong increase in the sample stiffness (see Figure S2c). However, a zoom-in into this curve shows that a weak effect brought about by the second cross-linking procedure is noticeable.

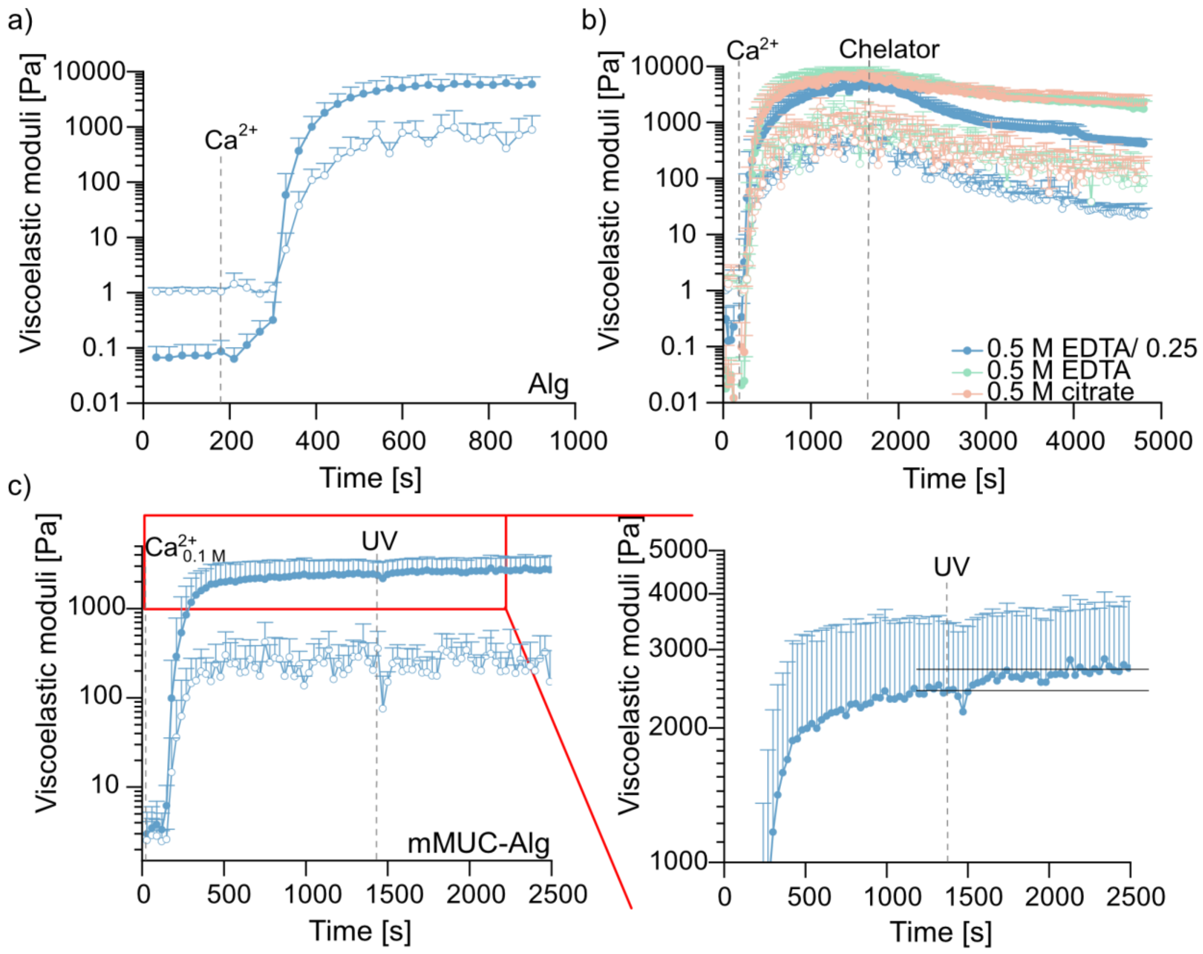

Figure S2. Viscoelastic moduli of 4% (w/v) alginate samples cross-linked in situ with 0.1 M $CaCl_2$ (a). Afterwards, different chelators were added to the cross-linked sample in situ: 0.5 M EDTA, 0.5 M citrate, and a combination of 0.5 M EDTA and 0.25 M citrate (b). When investigating a mixed system of mMUC-Alg, UV cross-linking conducted after ionic cross-linking does not lead to a strong increase in stiffness (c). The data shown represents mean values, error bars depict the standard deviation as calculated from the *n* = 3 samples.

Section S4: Ionic cross-linking of mucin

To assess if $CaCl_2$ can also induce ionic cross-links in networks of purified mucin MUC5AC, we added 50 mM $CaCl_2$ to a 1% (w/v) mucin solution and evaluated the ensuing change in the viscoelastic properties (see Figure S3). This $CaCl_2$ concentration was chosen since Rulff *et al.*[10] found that this concentration is optimal for cross-linking of bovine submaxillary mucin (MUC5B). However, for the porcine gastric mucin MUC5AC used here, we only observe a mild effect: upon Ca^{2+} addition, the mucin network is still very soft (with viscoelastic moduli below 1 Pa) and just at the transition point between a viscoelastic solution and a gel.

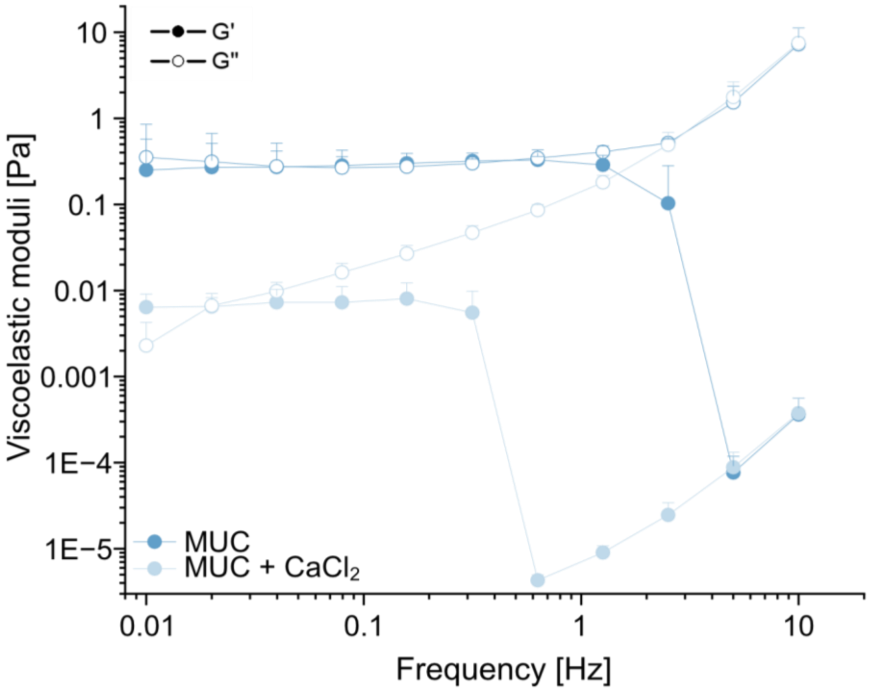

Figure S3. Viscoelastic moduli of 1% (w/v) mucin samples with and without the addition of 50 mM $CaCl_2$. Data shown represents mean values, error bars depict the standard deviation as calculated from $n = 3$ samples. If no error bars are visible, they are smaller than the symbol size.

Section S5: Homogeneity of mixed alginate/mucin samples

The homogeneity of the mixed alginate/mucin systems was analyzed by fluorescently labeling one of the two biopolymer components and imaging the labelled networks on a light microscope (see Figure S4).

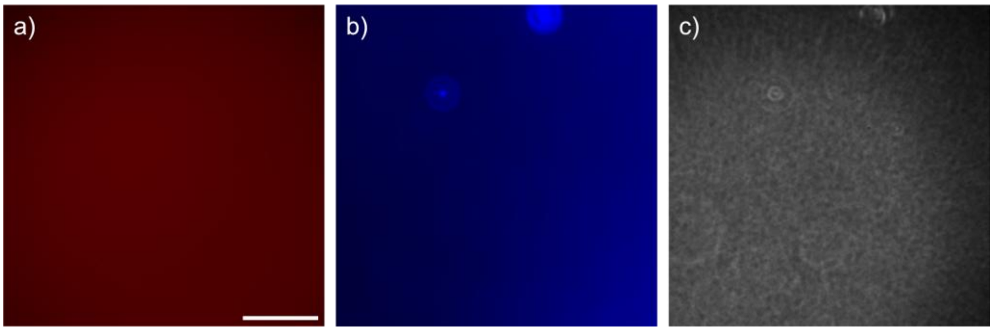

Figure S4. Microscopy images of a mixed mAlg/MUC network (2% (w/v) mAlg in combination with 2% (w/v) MUC). Fluorescence images of the ATTO 425-labeled mAlg (a) and the ATTO 594-labeled MUC components (b) are shown together with a phase contrast image (c). The scale bar represents 50 µm and applies to all three images.

Section S6: Dynamic change of stiffness in cycles

To test if it is possible to repeatedly switch the stiffness of a cross-linked alginate/mucin hydrogel system, we selected the mMUC-Alg system as an example and attempted to add and remove ionic cross-links several times in a row. For those measurements, the hole-y plate setup was combined with the UV transparent, custom-made measuring head, and a 0.01 M $CaCl_2$ solution was employed for initial cross-linking followed by UV exposure for secondary cross-linking. Afterwards, the chelator mix was introduced to remove the ionic cross-links, and this chelator mix was washed out with ddH_2O. Subsequently, the sample was cross-linked again, this time using a higher concentrated ion solution (0.1 M $CaCl_2$), which was followed by another chelator-induced un-linking step. The latter cross-linking/unlinking process was then repeated one more time. The experiment was performed with n = 3.

As shown in Figure S5 with our approach, a dynamic switch of hydrogel stiffness is even possible several times in a row. This demonstrates that, when combining covalent and ionic cross-links, the addition and removal of Ca^{2+} ions can be repeated at will.

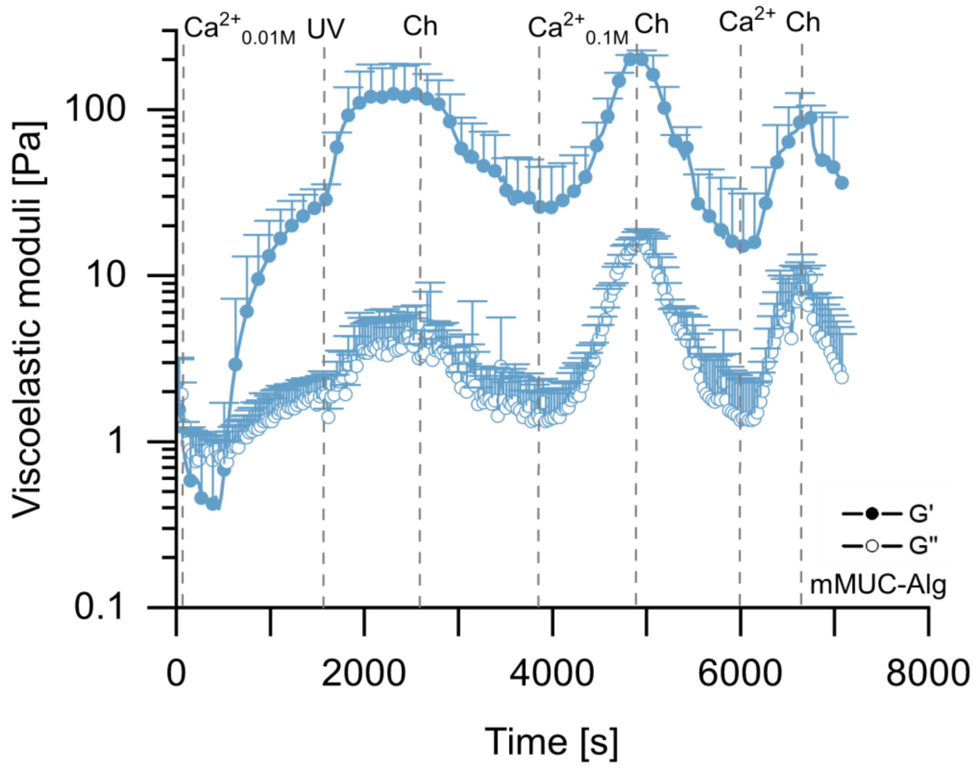

Figure S5. Dynamic tuning of the stiffness of a mMUC-Alg system (2% (w/v) each) cross-linked by both, ionic and covalent cross-links. Repeated addition of a chelator (Ch) solution and 0.1 M $CaCl_2$ solution removes/re-introduces ionic cross-links. Data shown represents mean values, error bars depict the standard deviation as calculated from *n* = 3 independent samples.

Section S7: Influence of the mixing ratio on the viscoelastic moduli of uncrosslinked mMUC/Alg samples

For the mixed alginate/mucin networks discussed in the main text, a mixture ratio of 1:1 ratio at an overall biopolymer concentration of 4% (w/v) was used. However, as depicted in Figure S6 for a mixture of alginate and mMUC, varying this mixture ratio can affect the stiffness of the system – even without introducing any cross-linking step.

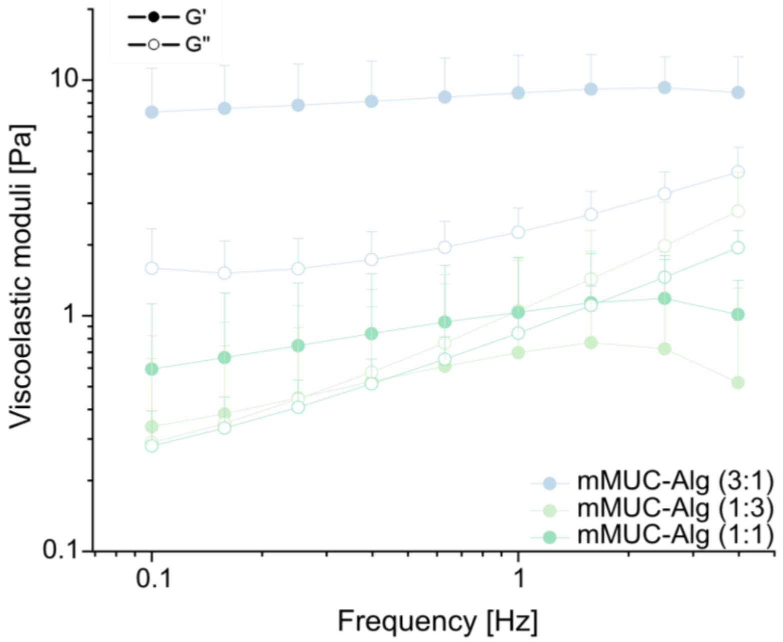

Figure S6. Viscoelastic frequency spectra of uncross-linked mMUC-Alg blends prepared at different mixture ratios. The data shown represents mean values, error bars depict the standard deviation as calculated from n = 3 samples.

Section S8: Degradation/swelling study

To investigate the stability of mucin/alginate hydrogels over time, two variants of mMUC-mAlg (2% (w/v) each) samples containing the UV suitable photo initiator were prepared with a volume of 300 µL in cylindrical molds (diameter: 10 mm). The samples were then illuminated with UV light from above for 10 min to create covalent cross-links. Afterwards, one of the sample variants was completely immersed in a 0.1 M $CaCl_2$ solution for 20 min to introduce ionic cross-links. To allow for the diffusive entry of Ca^{2+} ions from all sides (to obtain efficient ionic cross-linking), after 7 min of incubation, a spatula was used to detach the hydrogel from the walls of the mold, and the hydrogels were detached from the bottom of the mold after additional 6 min of incubation. Then, the hydrogels were placed into cell inserts and stored in 6 well-plates. Empty inserts as well as the inserts carrying the hydrogel samples were weighed. Subsequently, 5 mL of cell culture medium were added into each well and the plates were incubated at 37 °C and 5% CO_2. At each following time point, the cell inserts were removed from the wells, attached water droplets were dried with wipes, and afterwards weighed to determine the change in wet weight of the hydrogel samples. This experiment was performed with n = 16 replicates per sample type.

Interestingly, making use of more than one cross-linking strategy does not only offer to dynamically tune the mechanical properties of the gels, it also increases their stability over time. Supporting Information Figure S7 shows that mMUC-mAlg hydrogels swell when incubated in cell culture medium, and this occurs for both gel variants studied in this context (one contains covalent cross-links only, whereas the other one contains ionic cross-links in addition to covalent ones). This finding agrees with reports from the literature on other alginate-based hydrogel systems[11]. Moreover, when the mMUC-mAlg system is stabilized with ionic and covalent cross-links at the same time, it does not exhibit a measurable degradation behavior during the time period tested here and maintains its shape. This observation is likely to be a result of the increased cross-linking density in this sample compared to the purely ionically cross-linked (and thus softer) hydrogel variant. Thus, it is important to realize that the choice of biopolymer, its degree of modification, as well as the level and type cross-linking implemented into the hydrogel sample will have an influence on the degradation behavior of the gel, which should also allow for tailoring this hydrogel property according to its desired application.

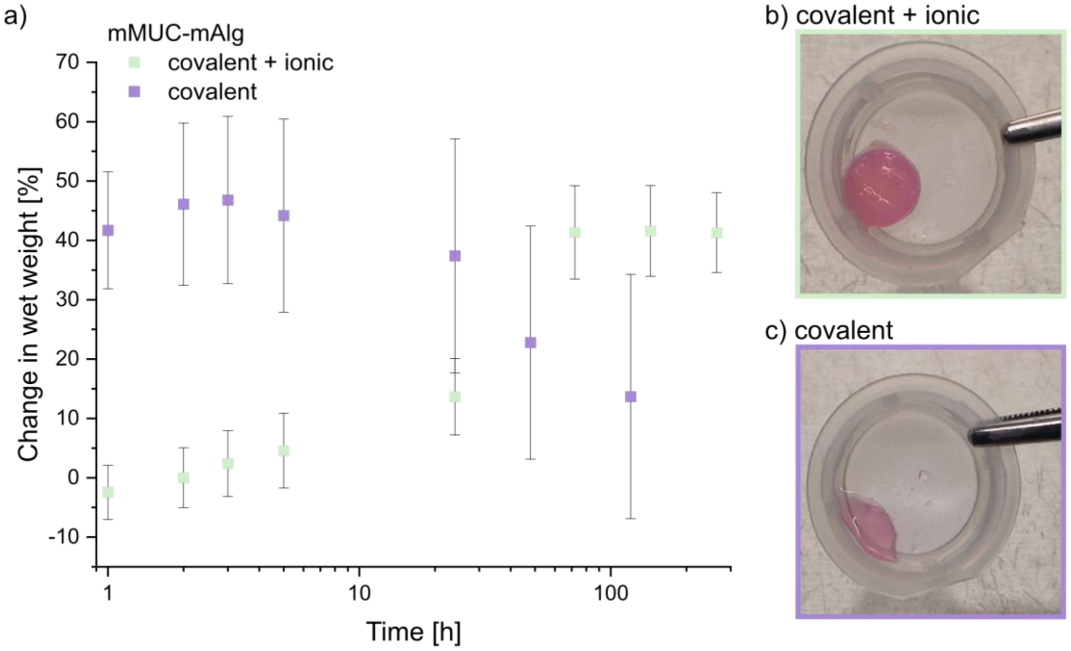

Figure S7. Degradation/swelling study of the mMUC-Alg (2% (w/v) each) hydrogel network, cross-linked covalently and ionically as well as only covalently. a) Change in wet weight [%] of the hydrogel samples. b) Representative image of the sample cross-linked both, ionically and covalently, after 11 days of incubation. c) Representative image of the sample cross-linked only covalently after 5 days of incubation.

Section S9: Effect of green light on cell viability

For biomedical applications, (bio)polymer cross-linking by exposure to green light can be a valuable alternative to UV induced cross-linking, as green light is reported to affect the viability of cells to a lower extent than UV light[12,13]. To complement the light exposure tests described in the main text, cell exposure tests towards UV and visible light were also analyzed 24 h after light exposure (see Figure S8a,b). Those experiments were motivated by our finding that cells exposed to UV light (analyzed 1 h after the illumination took place) already showed a round morphology (indicating some issues with viability) but still returned a bright green signal from the calcein staining (which indicates good metabolic activity). Nevertheless, they might have suffered from the light exposure process, and this might be observable when repeating the live/dead staining at later time points.

Representative microscopy images of such a later evaluation of cell viability are shown in Figure 8a, and their corresponding quantification is shown in Figure 8b. The low viability obtained for the control group (which is around 72 %) might be a result of the cells having been kept at room temperature for imaging on the previous day. It can be noted that, in contrast to cells illuminated with green light only (in the absence of a photo initiator), the other groups show a significant reduction in cell viability compared to the control group (which was not illuminated). Thus, green light seems to be favorable to achieve good levels of cell survival upon light exposure.

However, the incorporation of a photo initiator seems to reduce cell viability and thus can have a negative effect on the cells. This problem can be overcome by adjusting the illumination process (for representative microscopy images, see Figure 8c; the corresponding quantification is shown in Figure 8d). In addition, when using green light, the intensity of the light source as well as the total duration of the exposure process seem to impact the cell viability. By switching the light source off and on (as suggested by Etter *et al.* [13]), however, the cell viability remains high (*i.e.*, around ~70 %) even after one additional day of incubation. Thus, the process can be rated as cytocompatible to ISO 10993-5. Probably, this improved result originates from a reduced heat production caused by the lower light intensity or reduced exposure time.

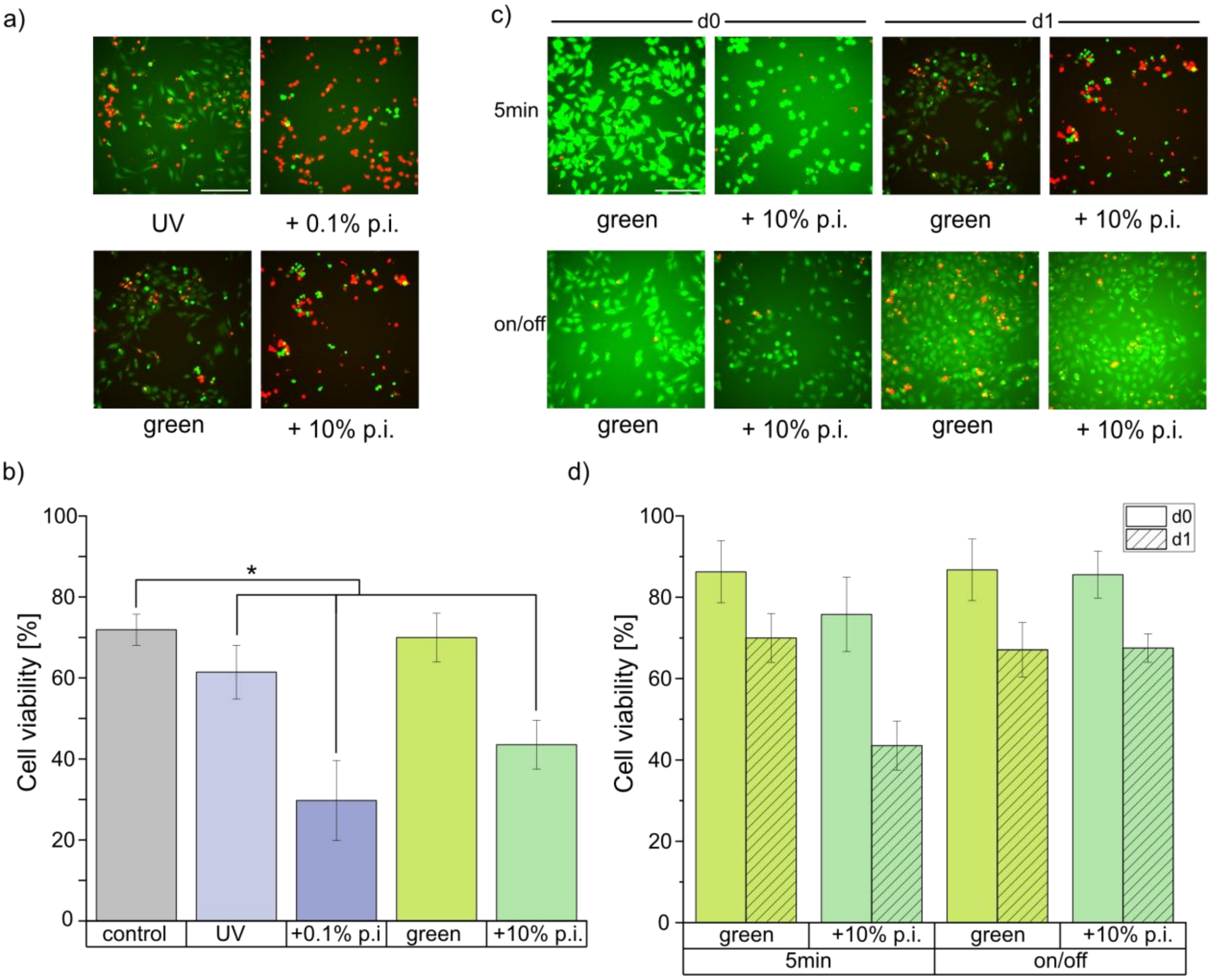

Figure S8. Additional viability experiments with HeLa cells to test for putative cytotoxic effects brought about by the hydrogel components or the chemicals/light exposure process used for cross-linking. a) Representative images of cells stained with a live/dead staining. The images were obtained 1 d after the incubation process with the different materials was conducted. b) Quantification of the images shown in a) and similar ones. c) Representative images of cells stained with a live/dead staining. The images were obtained directly (= 0 d) and 1 d after the cells were exposed to green light (by employing different illumination strategies) d) Quantification of the images shown in b) and similar ones. Data shown represents mean values, error bars depict the standard deviation as calculated from n = 5 samples (from which 3 images were obtained each). Asterisks denote statistically significant differences based on a p-value of 0.05. The scale bars in the microscopy images represent 200 µm and apply to all images shown in this figure.

Section S10: Molecular weight determination of alginate

The molecular weight of the alginate variant used here was determined by combining measurements of refractive indices (conducted with a digital refractometer, DR201 95, 589 nm, Krüss GmbH, Hamburg, Germany) with light scattering tests (conducted on a particle size analyzer, Litesizer 500, Anton Paar, Graz, Austria)[7]. First, the refractive indices *n* of different alginate solutions (reconstituted in water at different concentrations *c* of 2.5, 5.0, and 10 mg/mL; 154 mM NaCl was added to each solution) were determined (see Figure S9).

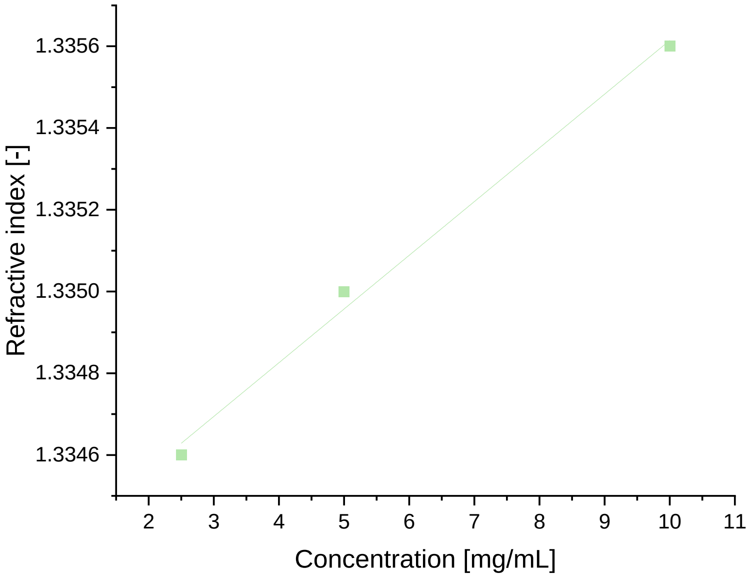

Figure S9. Averaged refractive index values obtained for different alginate solutions (*n* = 3 per data point).

Then, the d*n*/d*c* value obtained from those measurements (d*n*/d*c* = 0.1314 mL/g; R^2 = 0.99436) was employed to determine the molecular weight. Here, static light scattering measurements were conducted for alginate solutions prepared at different concentrations (0.1, 0.5, and 1 mg/mL). The obtained molecular weight of alginate was M_w = (342 ± 7) kDa, which agrees with the literature[14].

Section S11: Degree of methacrylation

To analyze the efficiency of the methacrylation process, the degree of substitution was determined by conducting absorbance measurements (Varioskan LUX multimode microplate reader, Thermo Fisher Scientific) with modified and unmodified alginate/mucin solutions and comparing the obtained values to reults obtained from a dilution series of pure methacrylic anhydride (see Figure S10a and b). The standard curve obtained from the latter is shown in Figure S10c and has an R^2 value of 0.99677.

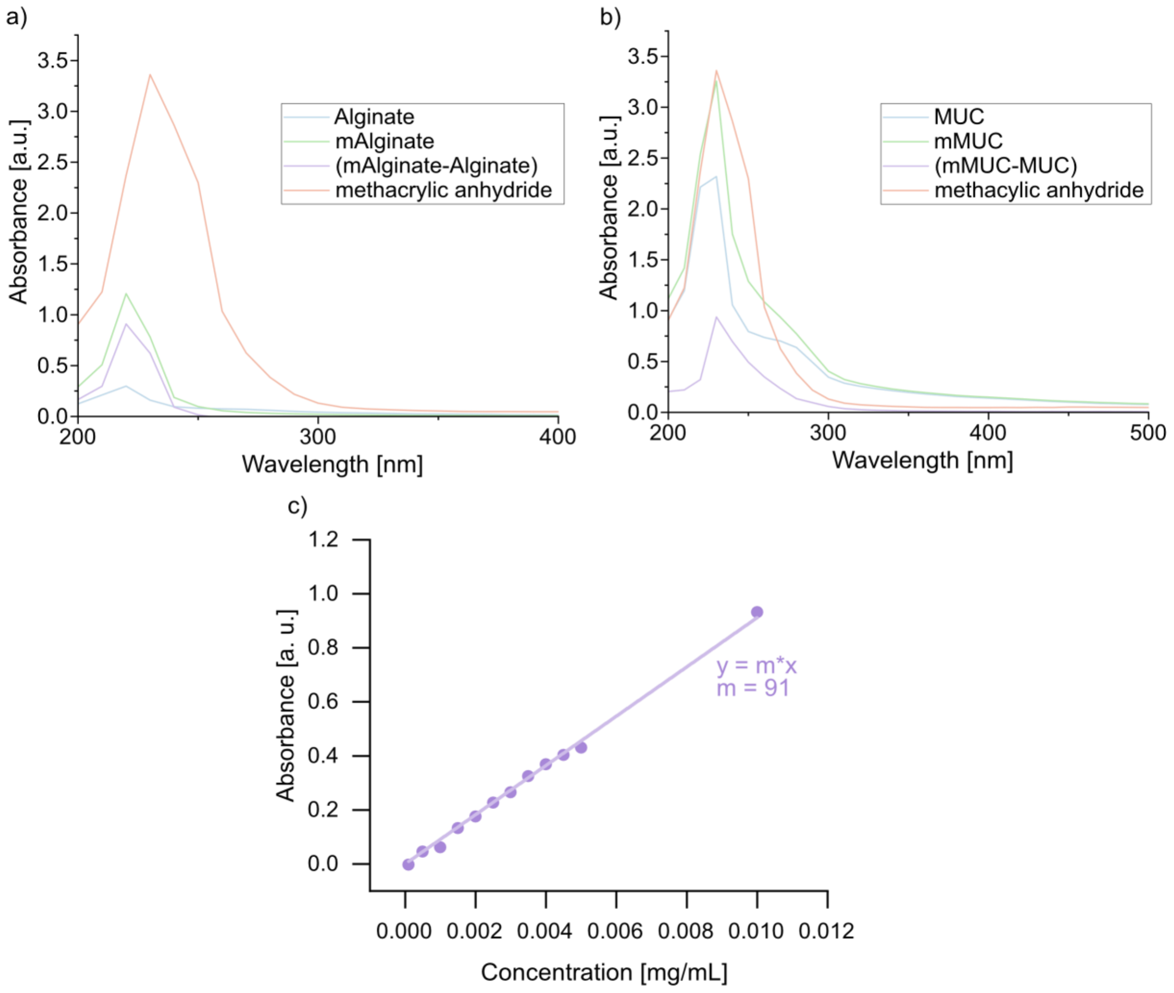

Figure S10. Absorbance spectra of (a) alginate-based samples, (b) mucin-based samples, and (c) the standard curve obtained for methacrylic anhydride solutions at 220 nm. (*n* = 3)

With those results, we calculated methacrylic anhydride concentrations of $2 \cdot 10^{-4}$ mg/mL for a solution of 0.01 % MUC and of $4 \cdot 10^{-4}$ % for a solution of 0.01 %

Alg. By considering the molecular weight of an MUC5AC oligomer to be ~9 MDa and the molecular weight of alginate to be 342 kDa (see above), we estimated the number of methacrylic anhydride groups per molecule to be around 26 for MUC and 18 for Alg.

Section S12: Conjugation efficiency of mucin with crDNA

To investigate the conjugation efficiency of crDNA to mucins, first, the fluorescent intensities of a dilution series of fluorescently labelled crDNA (Integrated DNA Technologies, Germany) generated in ultrapure water was measured using a microplate reader (Varioskan LUX multimode microplate reader, Thermo Fisher Scientific) at Ex/Em: 685/706 nm (see Figure S11).

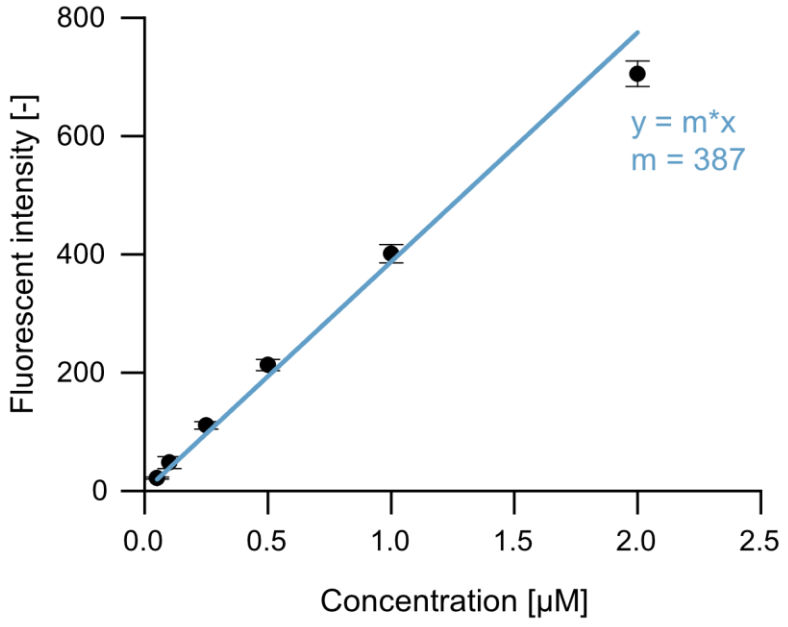

Figure S11. Concentration-dependent fluorescent intensities as obtained for fluorescently labeled crDNA. Data shown represents mean values, error bars depict the standard deviation as calculated from *n* = 3 samples.

Furthermore, a sample was prepared in which fluorescently labelled crDNA was coupled to mucins. This sample was dialyzed in desalted water for 7 h to remove unbound crDNA (MWCO: 12–14 kDa, Pure-A-Lyzer Maxi Dialysis Kit, Sigma-Aldrich, St. Louis, USA) and subsequently lyophilized. A 0.5 % (w/v) solution of this sample was

then dissolved in ultrapure water, and its fluorescent intensity was evaluated with a microplate reader (Varioskan LUX multimode microplate reader, Thermo Fisher Scientific) at Ex/Em: 685/706 nm[15]. From $n = 4$ replicates, an average value of 241.7 ± 5.9 was determined, which corresponds to a conjugation efficiency of around 8.3 %.

Section S13: Permeability of the filter membrane towards DNA strands

The hole-y plate setup allows for the addition of cross-linking agents as well as chelators *in situ* and during the measurement. Here, a membrane creates an interface between the sample and the liquid reservoir located in the bottom plate (to avoid a loss of polymeric material during the measurements). Before conducting decross-linking tests with dDNA, the permeability of this membrane towards DNA strands was tested. Therefore, the membrane was inserted into filter holders (CellCrown 96 inserts, Scaffdex, Tampere, Finland) and a known concentration of dDNA prepared in an aqueous solution containing 0.1 mM EDTA was added on top of the membrane. After 0.5 h and 23NanoDrop-1000 spectral photometer (Thermo Fischer Scientific, Ulm, Germany),

Table S3. Concentration of dDNA after diffusion through the membrane after 0.5 h and 23 h of incubation at room temperature and the percentage of dDNA that successfully diffused through the membrane. Data shown represents mean values as calculated from $n = 7$ (for 0.5 h) and $n = 5$ (for 23 h) samples.

Time (h)	Concentration (ng/µL)	Percentage of starting concentration (%)
0.5	18 ± 5	58
23	22 ± 2	73

Section S14: Artefacts created by the pump during measurements with crDNA

Owing to the very soft characteristics of crDNA cross-linked hydrogels, the parameters chosen for the time-dependent rheological measurements were adapted to a frequency of 0.2 Hz and a torque of 0.1 µNm to ensure proper detection of the changing viscoelastic properties. For those very soft samples, artefacts were recorded when the pump was switched on (see Figure S12) to inject either Ca^{2+} ions or the chelator mixture into the reservoir of the hole-y plate. However, those artefacts were only transient and did not affect more than 1 or 2 data points. To be able to calculate a reliable average from those curves, those few data points affected by this artefact were removed from the curves shown in the main text.

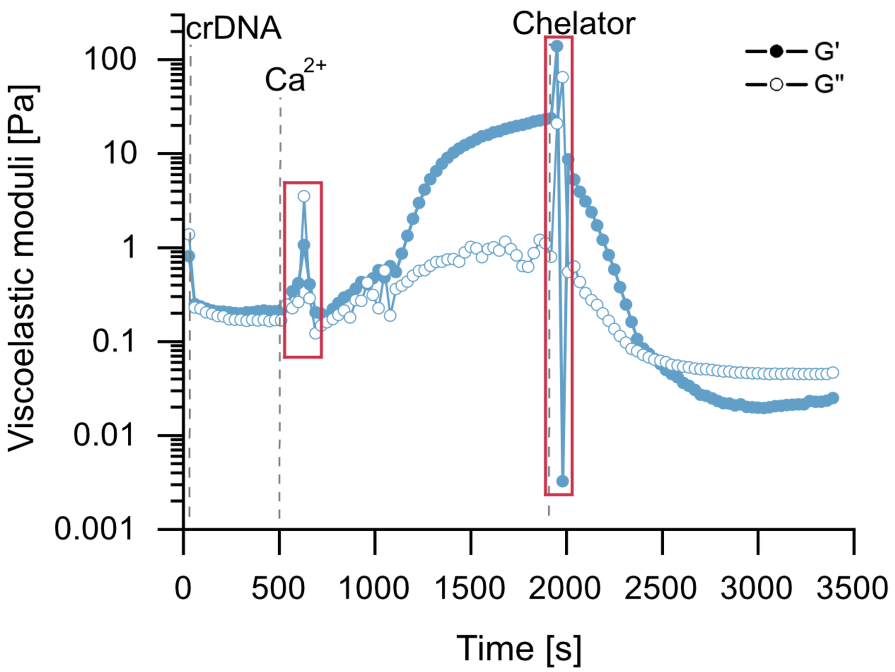

Figure S12. Example curve illustrating the influence of switching on the pump during a measurement conducted with a very soft, crDNA cross-linked hydrogel. Data points shown in red boxes were affected by the artefact created by the pump activation. (n = 1)

Section S15: Bacterial strains and media

The bacterial strains tested in this study were obtained from the following suppliers: Institute Pasteur, France (*Pseudomonas aeruginosa* PAO1), American Type Culture Collection ATCC, USA (*Streptococcus pyogenes* ATCC 700294, *Staphylococcus aureus* USA300 Lac (J). *Escherichia coli* 536 was provided by the strain library of the Chair of Organic Chemistry II (TUM).

For the cultivation of those bacteria, the following media were used:

- for *S. aureus*: B medium (Lysogeny broth with 0.1 % K_2HPO_4, 10 g/L casein peptone, 5 g/L NaCl, 5 g/L yeast extract, 1 g/L K_2HPO_4, pH 7.5);
- for *E. coli* and *P. aeruginosa*: LB medium (Lysogeny broth, 10 g/L casein peptone, 5 g/L NaCl, 5 g/L yeast extract, pH 7.5);
- for *S. pyogenes:* BHB medium (Brain-heart-infusion broth, 7.5 g/L brain infusion, 10 g/L heart infusion, 10 g/L peptone, 5 g/L NaCl, 2.5 g/L Na_2HPO_4, 2 g/L glucose, pH 7.4).

Agar plates were made using 1.5 % agar solutions supplemented with the appropriate medium.

Cited Literature

(1) Tordi, P.; Ridi, F.; Samorì, P.; Bonini, M. Cation-Alginate Complexes and Their Hydrogels: A Powerful Toolkit for the Development of Next-Generation Sustainable Functional Materials. *Adv. Funct. Mater.* **2024**, *35* (9). DOI: 10.1002/adfm.202416390
(2) Marczynski, M.; Jiang, K.; Blakeley, M.; Srivastava, V.; Vilaplana, F.; Crouzier, T.; Lieleg, O. Structural Alterations of Mucins Are Associated with Losses in Functionality. *Biomacromolecules* **2021**, *22* (4), 1600-1613. DOI: 10.1021/acs.biomac.1c00073
(3) Wang, J.; Zhuang, S. Chitosan-based materials: Preparation, modification and application. *J. Clean. Prod.* **2022**, *355*. DOI: 10.1016/j.jclepro.2022.131825
(4) Xie, D.; Bian, J.; Ni, C.; Zhao, P.; Pu, Z.; Yue, J. Tuning Room-Temperature Injectability of Gelatin-Based Hydrogels via Introduction of Competitive Hydrogen Bonds. *ACS Macro. Lett.* **2025**, *14* (3), 313-319. DOI: 10.1021/acsmacrolett.5c00018
(5) Rather, J. A.; Akhter, N.; Ashraf, Q. S.; Mir, S. A.; Makroo, H. A.; Majid, D.; Barba, F. J.; Khaneghah, A. M.; Dar, B. N. A comprehensive review on gelatin: Understanding impact of the sources, extraction methods, and modifications on potential packaging applications. *Food Packag. Shelf Life* **2022**, *34*. DOI: 10.1016/j.fpsl.2022.100945
(6) Salih, A. R. C.; Farooqi, H. M. U.; Amin, H.; Karn, P. R.; Meghani, N.; Nagendran, S. Hyaluronic acid: comprehensive review of a multifunctional biopolymer. *FJPS* **2024**, *10* (1). DOI: 10.1186/s43094-024-00636-y
(7) Gürer, U.; Mansi, S.; Reuter, M.; Arcuti, D.; Hadzhieva, Z.; Günsel, U.; Hagn, F.; Boccaccini, A. R.; Mela, P.; Lieleg, O. Cellulose-based bilayer films with asymmetric properties for the sealing of tissue lesions. *Cellulose* **2025**. DOI: 10.1007/s10570-025-06486-y
(8) Dang, X.; Li, N.; Yu, Z.; Ji, X.; Yang, M.; Wang, X. Advances in the preparation and application of cellulose-based antimicrobial materials: A review. *Carbohydr. Polym.* **2024**, *342*, 122385. DOI: 10.1016/j.carbpol.2024.122385
(9) Li, S.; Dan, X.; Chen, H.; Li, T.; Liu, B.; Ju, Y.; Li, Y.; Lei, L.; Fan, X. Developing fibrin-based biomaterials/scaffolds in tissue engineering. *Bioact. Mater.* **2024**, *40*, 597-623. DOI: 10.1016/j.bioactmat.2024.08.006
(10) Rulff, H.; Schmidt, R. F.; Wei, L. F.; Fentker, K.; Kerkhoff, Y.; Mertins, P.; Mall, M. A.; Lauster, D.; Gradzielski, M. Comprehensive Characterization of the Viscoelastic Properties of Bovine Submaxillary Mucin (BSM) Hydrogels and the Effect of Additives. *Biomacromolecules* **2024**. DOI: 10.1021/acs.biomac.4c00153
(11) Zhu, H.; Monavari, M.; Zheng, K.; Distler, T.; Ouyang, L.; Heid, S.; Jin, Z.; He, J.; Li, D.; Boccaccini, A. R. 3D Bioprinting of Multifunctional Dynamic Nanocomposite Bioinks Incorporating Cu-Doped Mesoporous Bioactive Glass Nanoparticles for Bone Tissue Engineering. *Small* **2022**, *18* (12), e2104996. DOI: 10.1002/smll.202104996
(12) Schwarz, T. UV light affects cell membrane and cytoplasmic targets. *J. Photochem. Photobiol. B: Biol.* **1998**, *44* (2), 91-96. DOI: 10.1016/S1011-1344(98)00126-2
(13) Etter, J. N.; Karasinski, M.; Ware, J.; Floreani, R. A. Dual-crosslinked homogeneous alginate microspheres for mesenchymal stem cell encapsulation. *J. Mater. Sci. Mater. Med.* **2018**, *29* (9), 143. DOI: 10.1007/s10856-018-6151-4
(14) Olad, A.; Pourkhiyabi, M.; Gharekhani, H.; Doustdar, F. Semi-IPN superabsorbent nanocomposite based on sodium alginate and montmorillonite: Reaction parameters and swelling characteristics. *Carbohydr. Polym.* **2018**, *190*, 295-306. DOI: 10.1016/j.carbpol.2018.02.088

(15) Henkel, M.; Kimna, C.; Lieleg, O. DNA Crosslinked Mucin Hydrogels Allow for On-Demand Gel Disintegration and Triggered Particle Release. *Macromol. Biosci.* **2024**, *24* (4), e2300427. DOI: 10.1002/mabi.202300427
